# Brn3b regulates the formation of fear-related midbrain circuits and defensive responses to visual threat

**DOI:** 10.1371/journal.pbio.3002386

**Published:** 2023-11-20

**Authors:** Hyoseo Lee, Hannah Weinberg-Wolf, Hae-Lim Lee, Tracy Lee, Joseph Conte, Carlos Godoy-Parejo, Jonathan B. Demb, Andrii Rudenko, In-Jung Kim

**Affiliations:** 1 Department of Ophthalmology and Visual Science, Yale University School of Medicine, New Haven, Connecticut, United States of America; 2 Department of Cellular and Molecular Physiology, Yale University School of Medicine, New Haven, Connecticut, United States of America; 3 Department of Biology, City College of New York, New York, New York, United States of America; 4 Department of Neuroscience, Yale University School of Medicine, New Haven, Connecticut, United States of America; 5 Wu Tsai Institute, Yale University, New Haven, Connecticut, United States of America; 6 Graduate Programs in Biology and Biochemistry, City University of New York, New York, New York, United States of America; Icahn School of Medicine at Mount Sinai Friedman Brain Institute, UNITED STATES

## Abstract

Defensive responses to visually threatening stimuli represent an essential fear-related survival instinct, widely detected across species. The neural circuitry mediating visually triggered defensive responses has been delineated in the midbrain. However, the molecular mechanisms regulating the development and function of these circuits remain unresolved. Here, we show that midbrain-specific deletion of the transcription factor Brn3b causes a loss of neurons projecting to the lateral posterior nucleus of the thalamus. Brn3b deletion also down-regulates the expression of the neuropeptide tachykinin 2 (Tac2). Furthermore, Brn3b mutant mice display impaired defensive freezing responses to visual threat precipitated by social isolation. This behavioral phenotype could be ameliorated by overexpressing Tac2, suggesting that Tac2 acts downstream of Brn3b in regulating defensive responses to threat. Together, our experiments identify specific genetic components critical for the functional organization of midbrain fear-related visual circuits. Similar mechanisms may contribute to the development and function of additional long-range brain circuits underlying fear-associated behavior.

## Abstract 2

Fear responses to threat represent an essential survival instinct. While previous work has focused primarily on the forebrain and limbic system, this study examines the midbrain visual circuitry, identifying specific molecular mechanisms underlying its organization and fear-related behavioral function.

## Introduction

Behavioral responses to threat are crucial for both animal and human survival. In humans, the inaccurate interpretation of threat- and fear-related information can lead to devastating psychiatric conditions [1–4]. Threatening stimuli are detected by sensory systems, including the visual system. Visually triggered defensive fear responses depend on midbrain structures, including the superior colliculus (SC) and periaqueductal gray (PAG) [5–7]. Threatening visual signals are conveyed to the superficial SC and subsequently delivered to the PAG either directly via the deep SC or indirectly within circuits connecting SC to other subcortical areas that ultimately project to the PAG [8–10]. The PAG integrates threat-related information and executes relevant defensive reactions. Specific midbrain circuits mediating defensive behaviors to visual threat have been identified using electrical and neurochemical stimulation as well as optogenetic and chemogenetic tools [7–14]. However, these studies evaluated rather broadly defined cell populations (e.g., based on the expression of CaMKIIa, parvalbumin, and VGluT2) leaving a gap in our understanding of the molecular mechanisms underlying the organization and functional specificity of the identified circuits.

To examine molecular mechanisms that control development of the circuits mediating visually triggered fear responses, we sought genes expressed by subsets of midbrain neurons. We focused on the superficial SC, which conveys visual information from the retina and visual cortex to other subcortical areas [15], and discovered the expression of the transcription factor *Brn3b* within the bottom layer of superficial SC. Neurons in this layer innervate the lateral posterior nucleus (LP) of the thalamus, and SC-LP connections apparently mediate innate fear responses to visual threat manifested by freezing behavior [9,10,14]. We also discovered *Brn3b* expression confined to the deep SC and PAG. The overall expression pattern of *Brn3b* led us to hypothesize that this transcription factor may play important roles in the development of subcortical circuits critical for fear-related defensive behaviors.

Previous studies demonstrated a critical role for *Brn3b* in retina development [16,17], but little is known about *Brn3b* functions in other parts of the nervous system. Here, we report multifaceted roles of *Brn3b* in the organization and function of visual threat-related circuits, involving both superficial SC and deep SC/PAG. Conditional deletion of *Brn3b* in the midbrain decreased axonal projections to the LP due to neuronal loss in the superficial SC and also decreased expression of the neuropeptide tachykinin 2 (*Tac2*) in the deep SC/PAG. *Brn3b* mutants exhibited diminished freezing responses to a threatening looming stimulus [18] following single housing-based social isolation. This behavioral impairment was rescued by overexpression of *Tac2* in the deep SC/PAG. These findings suggest that *Tac2* acts downstream of *Brn3b* and that a change of *Tac2* expression plays an important role in behavioral alterations in *Brn3b* mutants. Altogether, our findings define, for the first time, the molecular mechanism that regulates development and function of midbrain circuits conveying defensive responses to visual threat.

## Results

### Layer-restricted Brn3b expression in the dorsal midbrain

**Fig 1:**
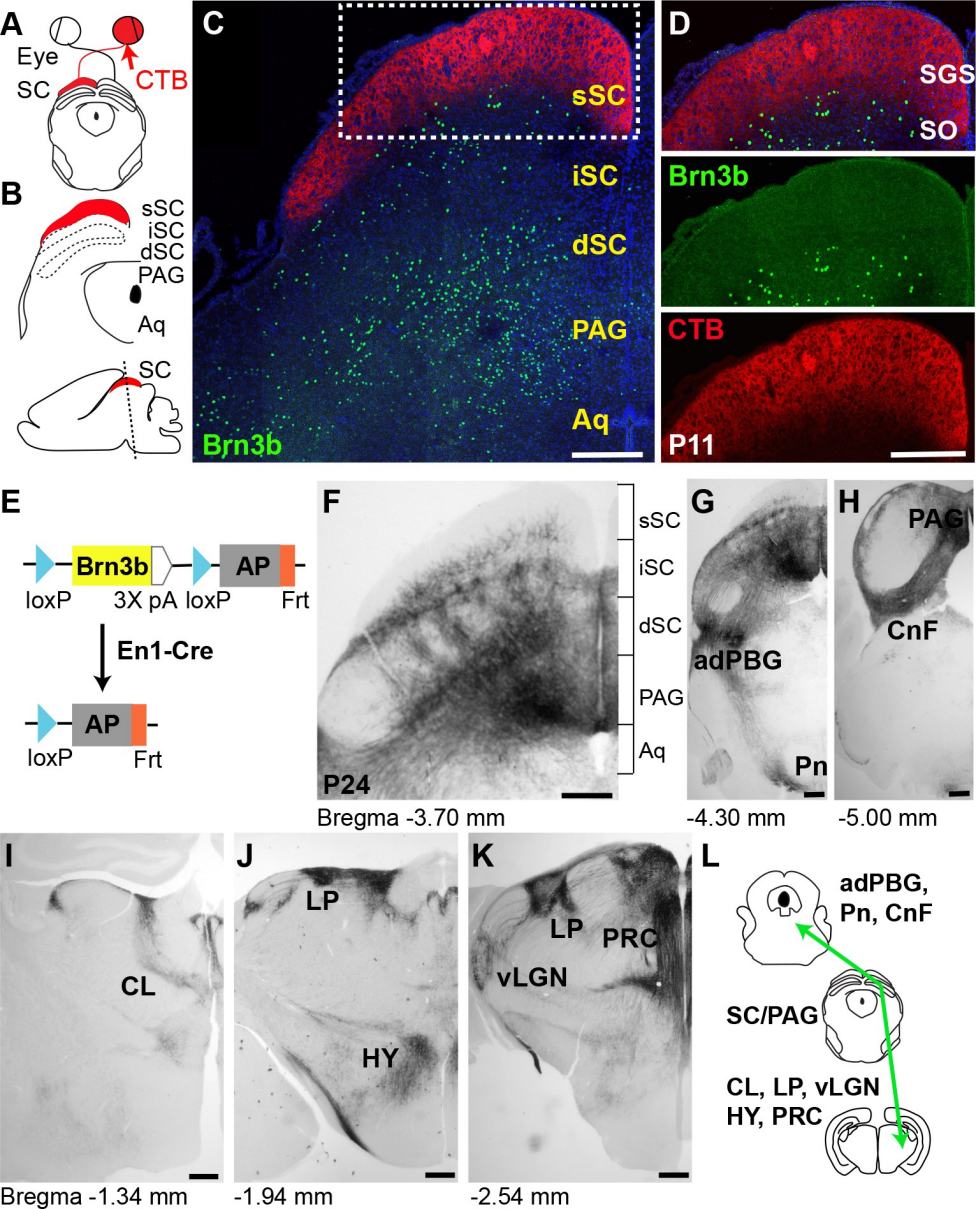
Layer-restricted Brn3b expression in the dorsal midbrain. ** (A, B)** Schematic diagrams of the midbrain after CTB injection into the contralateral eye (**A**); detailed layer distribution in the coronal section of the dorsal midbrain (top) and a sagittal image showing the location (dotted line) of the imaged coronal section (bottom) (**B**). **(C, D)** Brn3b expression (green), visualized by anti-Brn3b antibody is detected in the sSC, iSC, dSC, and PAG at P11 (**C**). Magnified view of the boxed area (**D**) shows Brn3b expression confined to the bottom layer of the sSC (SO). CTB labeling (red) divides the superficial SC into the SGS and SO. The aqueduct (Aq) is also shown. DAPI (blue). **(E)** Schematic diagram of the conditional *Brn3b* allele. Conditional deletion (by *En1-Cre*) removes an open reading frame of *Brn3b* and places a human placental AP under the control of the *Brn3b* promoter. **(F)** Dorsal midbrain section from *Brn3b*^*flox/+*^:: *En1-Cre* mouse histochemically processed for AP signals at P24. Brackets represent the layer segregation. **(G-K)** Axonal projections from the dorsal midbrain to adPBG and Pn **(G)**, CnF **(H)**, central lateral nucleus of the thalamus (CL) **(I)**, lateral posterior nucleus (LP) and hypothalamus (HY) **(J)**, vLGN and PRC **(K)**. Each area was identified by its anatomical position (see Methods). **(L)** Schematic diagram of the midbrain Brn3b+ neuronal projections. Scale bars: 250 μm. adPBG, adjacent parabigeminal nucleus; AP, alkaline phosphatase; CnF, cuneiform nucleus; CTB, cholera toxin B; dSC, deep SC; iSC, intermediate SC; PAG, periaqueductal gray; Pn, pons; PRC, precommisural nucleus; SC, superior colliculus; SGS, stratum griseum superficiale; SO, stratum opticum; sSC, superficial SC; vLGN, ventral lateral geniculate nucleus.

Our initial screening for SC neuronal markers in the superficial SC showed that Brn3b is expressed at the bottom layer of the superficial SC [15]. The organization of superficial SC can be defined relative to the axonal projections of retinal ganglion cells (RGCs). To visualize RGC axons, cholera toxin B subunit (CTB)-conjugated fluorescent dyes were delivered into 1 eye (Fig 1A–1C). CTB labeling divides superficial SC into 2 layers: stratum griseum superficiale (SGS), a densely labeled top layer, and stratum opticum (SO), a weakly labeled bottom layer. CTB labeling verified that Brn3b+ neurons, identified by immunostaining are localized in SO (Fig 1D). Furthermore, Brn3b is expressed sparsely in the intermediate SC and abundantly in the deep SC/PAG.

Neurons in specific layers of the dorsal midbrain selectively innervate different subcortical areas [19,20]. Restricted expression of Brn3b, particularly in the bottom layer of superficial SC (SO), prompted us to examine axonal projections of Brn3b+ neurons to other brain areas. To trace Brn3b+ axonal projections, we used a conditional *Brn3b* mouse carrying the *Brn3b* gene flanked by loxP sites and a human placental alkaline phosphatase (AP) coding region inserted immediately downstream of 3′ loxP (*Brn3b*^flox/+^, Fig 1E) [21]. Following Cre-mediated recombination, a copy of *Brn3b* is deleted and AP is expressed under the control of the *Brn3b* promoter. To visualize Brn3b+ neurons, we crossed *Brn3b*^flox/+^ to the *En1-Cre* line, in which Cre expression is confined to the midbrain [22,23]. The layer-restricted distribution of AP staining in the SC matched the labeling from the Brn3b antibody (S1A–S1G Fig): both AP and immunostaining signals in the superficial SC were detected close to the pia at P1 when SGS and SO are barely distinguishable, and then became restricted to the SO at P10/P12 when superficial SC development was completed [24]. Both methods revealed abundant labeling of somas/nuclei in the deep SC/PAG at P1. These observations confirmed that AP signals report Brn3b expression.

We next examined axonal projections to other brain areas, based on AP signals, and found that Brn3b+ neurons clearly innervate several subcortical areas, including LP, ventral lateral geniculate nucleus (vLGN), precommisural nucleus, hypothalamus, central lateral nucleus of the thalamus, adjacent parabigeminal nucleus, pons, and cuneiform nucleus (Fig 1F–1L). To rule out the possibility that observed axonal projections originate from areas outside the midbrain, we first conducted immunostaining of the thalamus including LP, and found no Brn3b staining, excluding the contribution of local thalamic neurons to the detected AP signals (S1H and S1I Fig). Considering the broad expression of Brn3b in the retina, we also examined *Brn3b*^flox/+^:: *En1-Cre* mice to ensure that labeling was not present in the retina’s output neurons, RGCs. Indeed, no AP signals were detected in the retina, confirming the midbrain-restricted Cre expression in the *En1-Cre* mouse (S1J Fig). Together, these results demonstrate that the observed axonal labeling represents Brn3b+ neuronal projections from the dorsal midbrain.

Next, we examined whether Brn3b+ neurons are glutamatergic or GABAergic, considering that SC projection neurons comprise both populations [9,25]. We initially attempted to mark glutamatergic and GABAergic neurons by either immunostaining or crossing *Slc17a6-Cre* (VGluT2-ires-Cre) or *Gad2-Cre* to a Cre-dependent td-Tomato reporter line (Ai14). However, very dense immunolabeling of synaptic terminals and overly abundant filling of neuronal processes by td-Tomato signals precluded visualizing individual somas. Instead, we performed in situ hybridization using *Slc17a6* and *Gad1* probes. The brain tissue for the hybridization was obtained from a newly generated *Brn3b*^GFP/+^ mouse, in which an open reading frame of *Brn3b* was replaced with *GFP* by CRISPR-based genome editing (S2A–S2C Fig). First, we confirmed that GFP signals faithfully represent Brn3b expression by double immunostaining, which showed that approximately 95% of Brn3b+ cells express GFP and approximately 93% of GFP+ cells express Brn3b. Subsequent double labeling with anti-GFP combined with in situ probes to either *Slc17a6* or *Gad1* revealed that all Brn3b+ neurons are glutamatergic and not GABAergic (S2D–S2L Fig).

### *Brn3b* is required for survival of neurons projecting to LP

To assess the role of *Brn3b* in circuit assembly of the midbrain, we crossed *Brn3b*^flox/flox^ to *Brn3b*^+/-^:: *En1-Cre* mice to generate *Brn3b*^flox/-^:: *En1-Cre* progeny (*Brn3b* cKO) and confirmed Brn3b loss in midbrain by immunostaining (S3A–S3E Fig). The conditional approach avoids confounding effects of retinal degeneration and, consequently, the compromised development of superficial SC in global *Brn3b* mutants [17,26]. Therefore, we proceeded to analyze and compare the phenotypes of control animals carrying conditional *Brn3b* and WT alleles (*Brn3b*^flox/+^:: *En1-Cre*) and mutants carrying conditional and null *Brn3b* alleles (*Brn3b*^flox/-^:: *En1-Cre*, cKO); in both cases, mice carry 1 copy of *En1-Cre* and 1 copy of *Brn3b*^flox^, producing AP signals.

**Fig 2:**
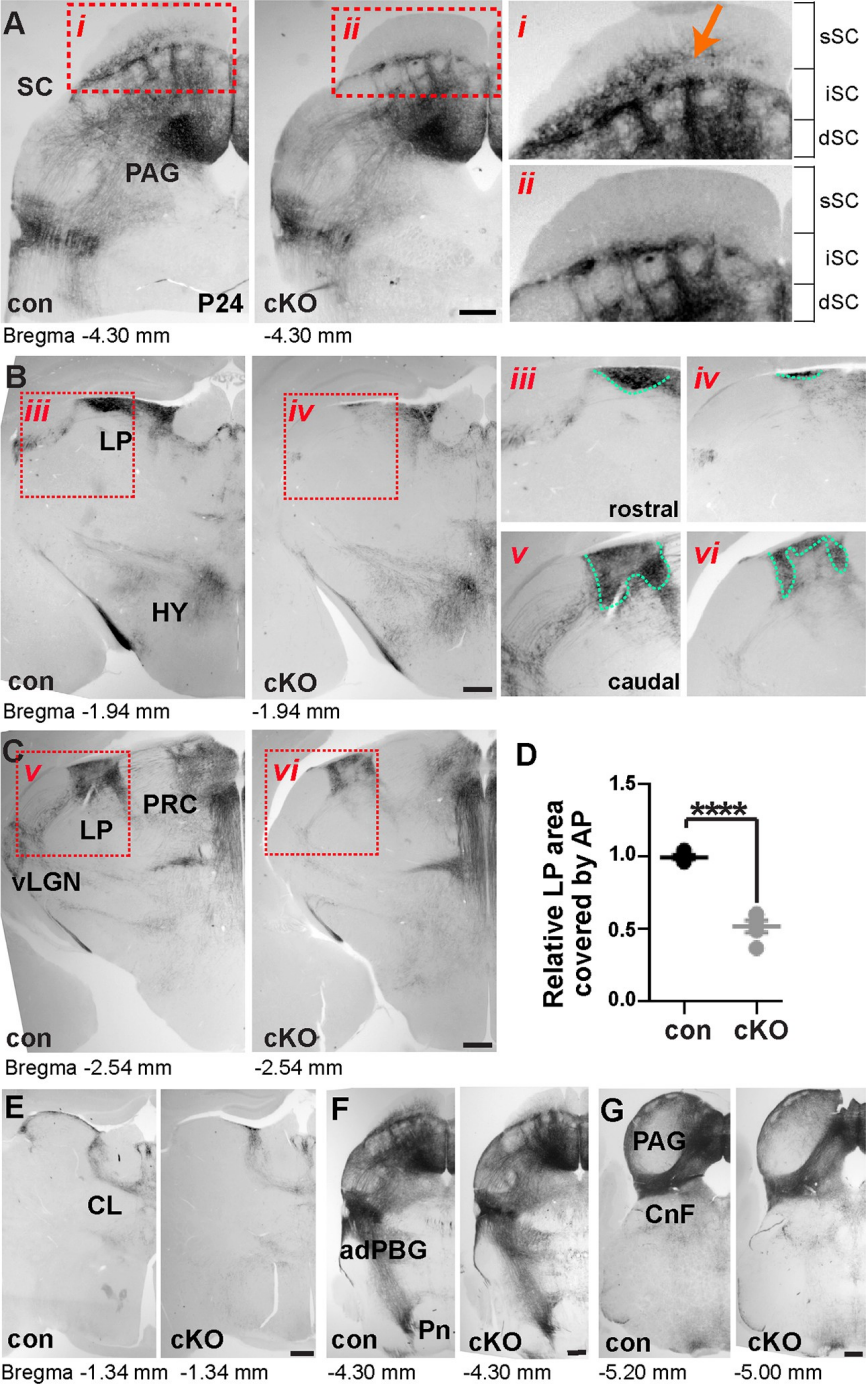
*Brn3b* deletion reduces the number of Brn3b+ neurons in the superficial SC and decreases projections to LP. Brn3b+ neurons visualized by AP signals. **(A)** (Left) Midbrain sections from control and conditional *Brn3b* knockout (cKO) at P24. (Right) Magnified view of the boxed areas (**i, ii**) showing the loss of Brn3b+ neurons in the superficial SC (arrow) of the cKOs. Brackets represent the layer distribution**. (B–D)** Decreased projections to the LP and vLGN and no difference in projections to the hypothalamus (HY) and PRC in cKO brains. Magnified view of the boxed images (**iii, vi**) showing the LP areas used for quantification (**D**; *n* = 5 mice/group). The AP-labeled LP area, normalized to the average control value, was reduced in cKO brains. Unpaired two-tailed Student’s *t* test (mean ± SEM, *p* < 0.0001 [****]). **(E–G)** No obvious differences in axonal projections to the central lateral nucleus of the thalamus (CL), adPBG, Pn, and CnF between control and cKO at P24 (*n* = 5 mice/group). Scale bars: 250 μm. The data underlying this figure can be found in S1 Data. adPBG, adjacent parabigeminal nucleus; AP, alkaline phosphatase; CnF, cuneiform nucleus; LP, lateral posterior nucleus; Pn, pons; PRC, precommisural nucleus; SC, superior colliculus; vLGN, ventral lateral geniculate nucleus.

The AP signals faithfully represent *Brn3b* expression in both control and cKO mice. These signals disappeared in the superficial SC of the mutants, with no obvious changes in other layers. Thus, *Brn3b* deletion induces a selective loss of Brn3b+ neurons in the superficial SC (Fig 2A). We also examined projections of Brn3b+ neurons to other brain areas and found that axonal projections to LP and ventral LGN were decreased in cKO mice, most significantly in the rostral LP and ventral LGN (Fig 2B–2D). Quantification of AP signals in the LP revealed an approximately 48% decrease. Axonal projections to other subcortical areas exhibited no obvious differences (Fig 2B and 2C and Fig 2E–2G).

The cKO animals lacked Brn3b+ neurons at the bottom layer of the superficial SC (SO), where LP-projecting neurons localize, which apparently explains the altered projections to LP. However, because some axonal projections to LP were still detectable in these cKO mice, we considered the possibility that Brn3b+ neurons in other layers of SC may also innervate LP. To test this idea, we first evaluated whether superficial SC Brn3b+ neurons project to LP using a second Cre line (S4A–S4D Fig). We crossed *Brn3b*^flox/+^ to *Ntsr1-GN209-Cre* mice, which express Cre in the LP-projecting superficial SC neurons [27,28], but we unexpectedly found AP signals also in the retina. However, projections of Brn3b+ neurons to LP persisted after enucleating both eyes, suggesting that labeled axons in LP originated from the SC and not from the retina. We also quantified the number of Brn3b+ neurons in the offspring of *Ntsr1-GN209-Cre* crossed to the Ai14 line expressing Cre-dependent td-Tomato. We found that approximately 23% of td-Tomato+ cells were Brn3b+. Interestingly, Brn3b mutants (*Brn3b*^flox/-^:: *Ntsr1-GN209-Cre*) showed no changes in the survival of the superficial SC Brn3b+ neurons and projections to the LP, likely because Cre expression occurs too late during development in this Cre line (see Discussion). Therefore, for further analysis, we utilized *En1-Cre*-based mutants only.

To confirm that Brn3b+ neurons in superficial SC project axons to LP, we labeled these neurons using 2 constructs delivered by adeno-associated virus (AAV): GFP-dependent FLP recombinase (FLP-DOG) and FLP-dependent mCherry (fDIO-mCherry). The FLP-DOG (DOG: Dependent On GFP) is genetically engineered to be unstable and degrade, whereas binding to GFP prevents its degradation [29]. Inside GFP+ cells, FLP-DOG becomes stabilized and converts fDIO-mCherry to an active configuration, allowing mCherry expression. We injected AAV-FLP-DOG and AAV-fDIO-mCherry into the superficial SC of *Brn3b*^GFP/+^ mouse to selectively label Brn3b+ neurons (S4E–S4I Fig). Double immunostaining revealed that approximately 98% of mCherry+ cells express GFP, suggesting high fidelity of the FLP-DOG method. Subsequent analysis revealed axons labeled with mCherry in LP, confirming that Brn3b+ neurons in superficial SC indeed innervate LP and that they are not local neurons. Lastly, we injected AAV-mCherry into the deep SC/PAG of wild-type mice and found axonal labeling in LP (S5A–S5D Fig). Together, our results indicate that LP is innervated by neurons of the superficial SC as well as deep SC/PAG and that *Brn3b* deletion by *En1-Cre* causes a loss of the superficial SC Brn3b+ neurons specifically, resulting in the decreased projection to the LP.

In addition to affecting the SC-LP connections, *Brn3b* deletion by *En1-Cre* completely eliminated innervation of the ventral LGN (Fig 2B and 2C). Given that the superficial SC Brn3b+ neurons project only to the LP and that delivering AAV-mCherry into the deep SC/PAG did not show projections to the ventral LGN, we speculated that the ventral LGN-projecting Brn3b+ neurons are located in the intermediate SC layer. To test this possibility, we delivered AAV-Cre into the intermediate SC of *Brn3b*^flox/+^ mice, which successfully labeled Brn3b+ neurons in the intermediate SC and confirmed their axonal projections to the ventral LGN (S5E–S5G Fig).

### *Brn3b* deletion-induced cell death occurs around birth

**Fig 3:**
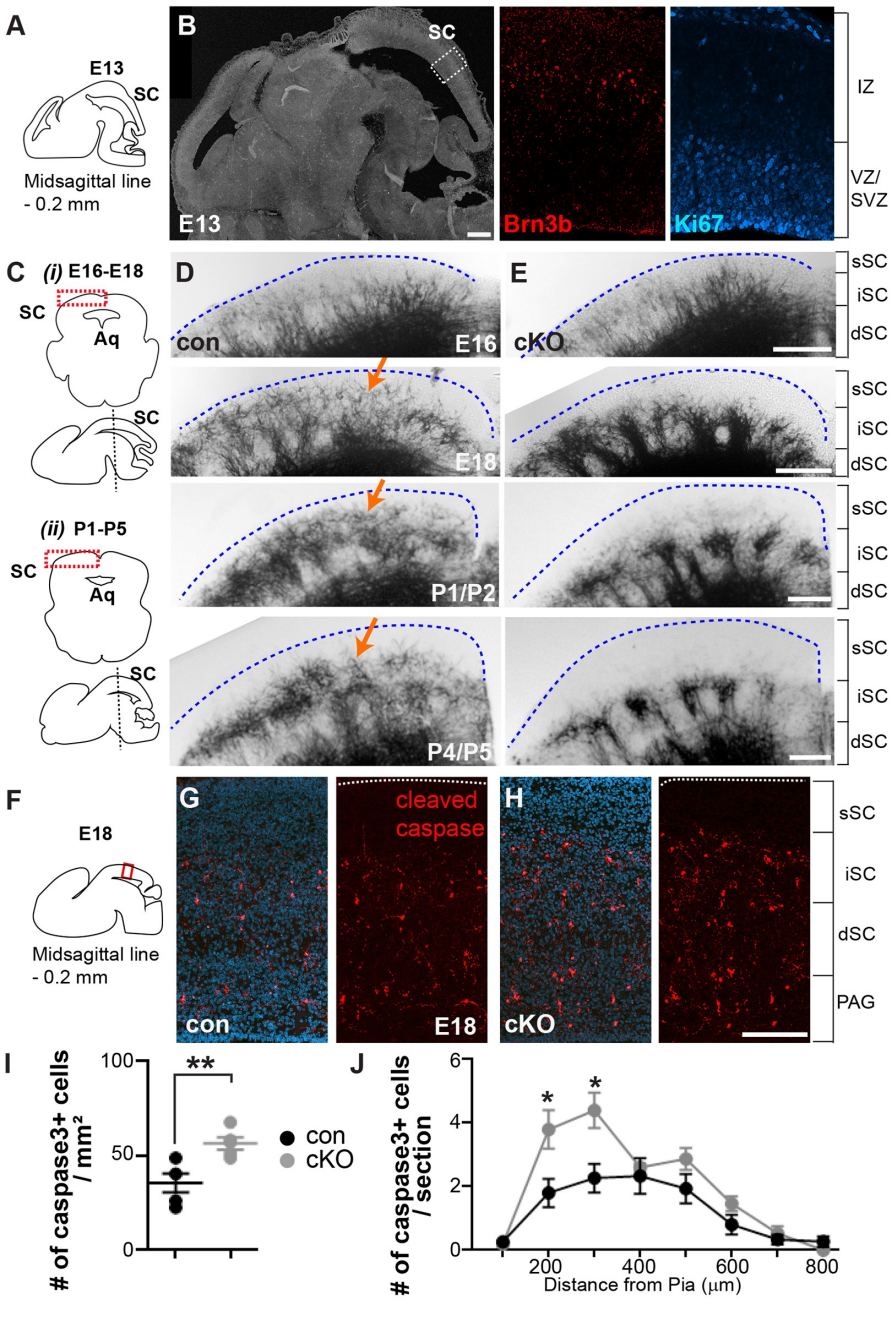
Loss of the superficial SC neurons in *Brn3b* mutants occurs around birth. **(A)** Schematic diagram illustrating a specific localization of the imaged sagittal section according to a lateral distance from the midsagittal line at E13. **(B)** (Left) Example of sagittal section. (Right) Magnified view of the boxed area. Brn3b+ neurons (red) were missing in the layer labeled by Ki67, a maker of proliferating cells (cyan) at E13 (*n* = 3 mice). **(C)** Schematic diagrams showing the brain area (boxed) of a coronal image used for analysis (top) and a sagittal image depicting the level (dashed line) where each coronal section was obtained (bottom) at E16-E18 (**i**) and at P1-P5 (**ii**). **(D, E)** Brn3b+ neurons, visualized by AP signals, were weakly detectable at E18 and become obvious at P1 and P4 (arrows) in the superficial SC of control (**D**). However, these neurons were missing at E18, P1 and P4 in the superficial SC of cKO (**E**) (*n* = 3 mice/group/development stage). **(F)** Schematic diagram depicting the brain areas (boxed) analyzed to examine the number of cleaved caspase-3+ cells in the sagittal section. The localization of imaged sections was shown as a lateral distance from the midsagittal line. **(G, H)** Cleaved caspase-3 immunoreactivity (red) revealed increased cell death at E18 in cKO (**H**), compared to control (**G**). DAPI (blue). **(I, J)** Quantification revealed increased cell death in cKO (35.7 ± 4.9/mm^2^ for control, 56.6 ± 3.3/mm^2^ for cKO; *n* = 5 mice/group), primarily occurring in the superficial and intermediate layers; unpaired two-tailed Student’s *t* test (mean ± SEM, *p* = 0.008 [**] in **I** and *p* = 0.03 [*] and 0.02 [*] in **J**). Brackets represent the layer segregation and dashed lines indicate the pial surface. Scale bars: 250 μm (**B**) and 150 μm (**D–E** and **G–H**). The data underlying this figure can be found in S1 Data. AP, alkaline phosphatase.

We next analyzed development of Brn3b+ neurons to define specific time points of neuronal loss and altered projections to LP. During development, Brn3b expression in the dorsal midbrain becomes detectable as early as E13 (Fig 3A and 3B). Therefore, we examined the possibility that Brn3b could regulate early development including neurogenesis. However, we found that Brn3b expression is barely detectable in the layer defined by a marker of proliferating cells, Ki67, suggesting that Brn3b is expressed post-mitotically and regulates later aspects of neuronal development and differentiation. We subsequently examined control mouse brains at later developmental time points, E16, E18, P1, and P4 (Fig 3C–3E). Based on the AP signals, segregation of Brn3b+ neurons into the superficial SC was first detectable at E18 and became obvious at P1. In cKO mice, the loss of superficial SC Brn3b+ neurons was already evident at E18 and persisted until adult stages (Fig 2A). It is known that *Brn3b* deletion causes apoptotic death of RGCs during retinal development [17]. To examine whether *Brn3b* deletion likewise causes apoptotic death of Brn3b+ neurons in the superficial SC, we performed immunostaining with anti-cleaved caspase-3 (Fig 3F–3J). Indeed, *Brn3b* deletion increased the number of cleaved caspase-3+ cells at E18 (approximately 59% increase in cKO) and this labeling was mainly detected in superficial and intermediate SC, suggesting that *Brn3b* expression is necessary for neuronal survival in these layers.

The lack of AP signals in the superficial SC of *Brn3b* mutants indicates the death of Brn3b+ neurons. However, extensive AP signals in other layers made it difficult to distinguish somas from processes, preventing visualization of individual Brn3b+ cells. To assess possible changes of Brn3b+ neurons in other layers, we utilized the molecular markers ETV1 and Brn3a. ETV1 is expressed in Brn3b+ neurons occupying the intermediate SC layer (S6A–S6G Fig). Double immunostaining showed that approximately 50% of Brn3b+ neurons were ETV1+ and approximately 49% of ETV1+ neurons were Brn3b+ at P2. Quantification of ETV1+ neurons at E16 and P2 revealed no difference between control and cKO mice, suggesting that *Brn3b* deletion has no effect on the development of Brn3b+/ETV1+ cells. We also examined Brn3b+ neurons expressing Brn3a (S6H–S6M Fig). Double immunostaining showed that approximately 60% of Brn3b+ neurons were Brn3a+ and approximately 45% of Brn3a+ neurons were Brn3b+ at P1 and that Brn3b+/Brn3a+ neurons are mainly located in deep SC/PAG. Quantification of Brn3a+ neurons at E15 and P1 also revealed no difference in their number and distribution in control and cKO mice, indicating that *Brn3b* deletion has no substantial effects on the development of Brn3b+/Brn3a+ neurons.

Considering that ETV1 and Brn3a are expressed in only about half of Brn3b+ neurons in their respective areas of co-expression (intermediate SC and deep SC/PAG), we also examined survival of Brn3b+ neurons using the *Brn3b*^GFP/+^ mouse, in which GFP expression faithfully reports Brn3b expression (S2 Fig). We crossed *Brn3b*^flox/flox^ to *Brn3b*^GFP/+^:: *En1-Cre* mice and confirmed *Brn3b* loss in *Brn3b*^GFP/ flox^:: *En1-Cre* offspring (*Brn3b-GFP* cKO) by immunostaining (S7 Fig). Quantification of GFP+ signals at E16 revealed no difference between *Brn3b*^GFP/+^ (control) and *Brn3b-GFP* cKO. Also, no changes in GFP+ signals were detected in deep SC/PAG at P1/P2. However, we found approximately 30% decrease of GFP+ cell number in superficial and intermediate SC, confirming that *Brn3b* deletion affects neuronal survival in these layers specifically. Collectively, these results suggest that the loss of *Brn3b* has no effect on neuronal survival in the layers of dorsal midbrain outside the superficial and intermediate SC, including the deep SC/PAG.

We also examined axonal development during several embryonic and postnatal stages. We observed no obvious differences between control and *Brn3b* mutants at E13, suggesting that *Brn3b* deletion had no effects on initial axonal growth (S8 Fig). Consistent with our observations at P24, no obvious changes of AP signals were detected in other brain areas at E13, E16, and P1/P2. Projections to LP became visible at P4/P5 in control and cKO mice (S9 Fig), indicating that *Brn3b* deletion causes no significant delay in axonal growth. However, despite seemingly unaffected developmental timing, projections to LP were clearly decreased in the mutants (approximately 80% decrease at P4/P5, approximately 60% at P8/P9, approximately 55% at P12/P13). Altogether, our findings suggest that *Brn3b* deletion in the midbrain causes decreased projections to the LP due to the loss of superficial SC Brn3b+ neurons occurring around birth.

### *Brn3b* regulates expression of *Tac2* in the deep SC/PAG

**Fig 4:**
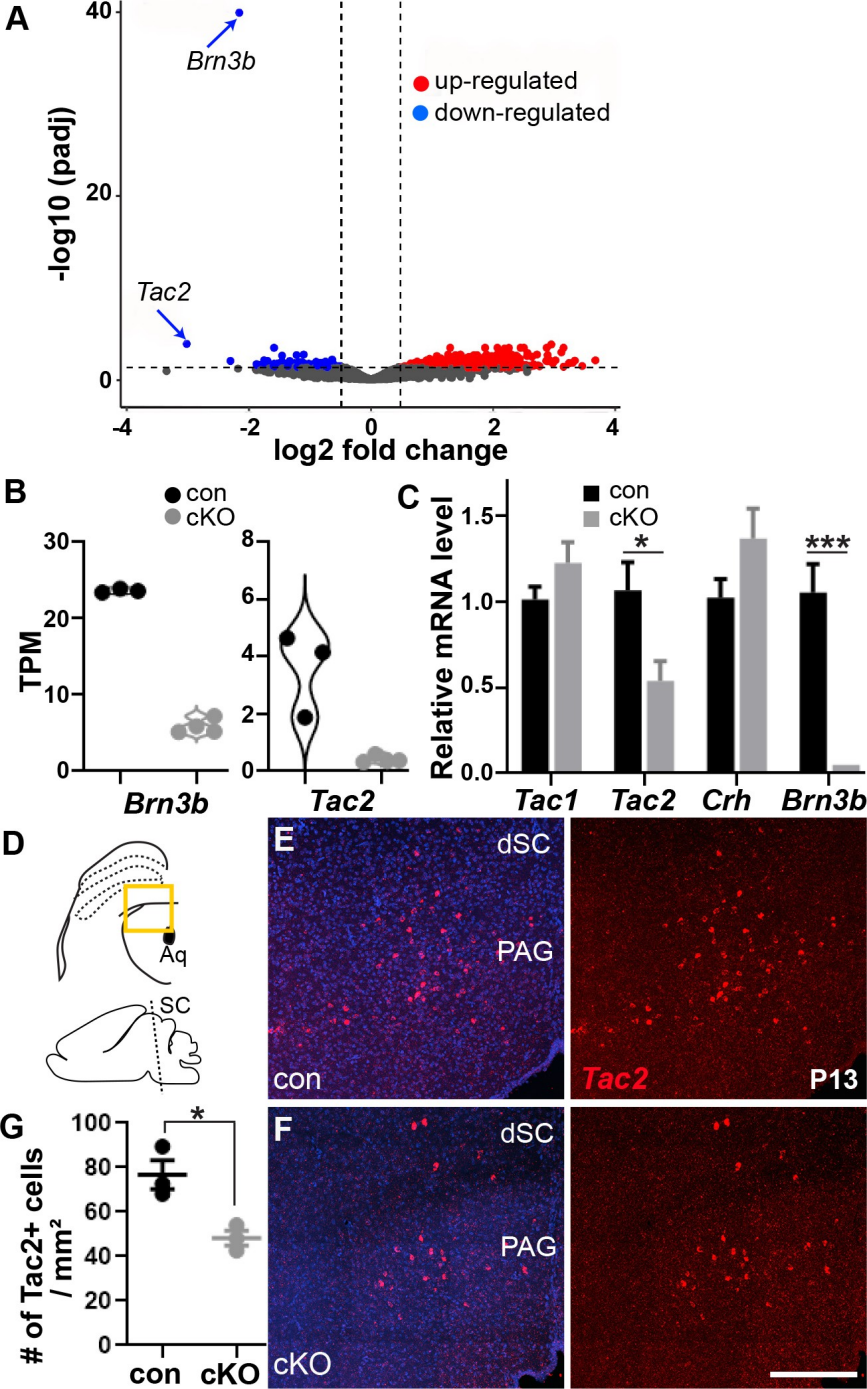
*Brn3b* regulates expression of *Tac2* in the deep SC/PAG. **(A, B)** Transcriptomic profiling (RNA-seq) using dorsal midbrain at P2-4 (*n* = 3 for control and 4 for cKO). **(A)** Volcano plot of the RNA-seq results. *Brn3b* and *Tac2* were down-regulated in cKO (arrows). The vertical dashed lines indicate the lowest log2-fold change value of significantly down- or up-regulated genes. The horizontal dashed line indicates adjusted *p* value (p_adj_) at 0.05. **(B)** Violin plots showing TPM values of *Brn3b* and *Tac2* for each animal. The log2-fold change was 2.01 for *Brn3b* and 3.10 for *Tac2*. **(C)** Assessment of *Tac1*, *Tac2*, and *Crh* levels at P3-P5 showed decreased expression of *Tac2* in cKO mice. The data for transcripts were normalized to the average control value. Quantification (*Tac1*: 1.01 ± 0.07 for control, 1.22 ± 0.12 for cKO; *Tac2*: 1.07 ± 0.16 for control, 0.54 ± 0.12 for cKO; *Crh*: 1.02 ± 0.11 for control, 1.37 ± 0.18 for cKO; *Brn3b*: 1.05 ± 0.17 for control, 0.01 ± 0.00 for cKO; *n* = 5 mice/group/gene). Unpaired two-tailed Student’s *t* test (mean ± SEM, *p* = 0.177 for *Tac1*, *p* = 0.030 [*] for *Tac2*, *p* = 0.136 for *Crh* and *p* = 0.0003 [***] for *Brn3b*). **(D)** Schematic diagram of the brain area analyzed for *Tac2* expression in a coronal section (top) and a sagittal image depicting the level (dashed line) where the coronal section was acquired (bottom). **(E, F)** *Brn3b* deletion decreased *Tac2* expression (red) in the deep SC/PAG, as detected by in situ hybridization. DAPI (blue). **(G)** Quantification of Tac2+ cell number (76.7 ± 6.5/mm^2^ for control, 48.2 ± 3.3/mm^2^ for cKO; *n* = 3 mice/group). Unpaired two-tailed Student’s *t* test (mean ± SEM; *p* = 0.017 [*]). Scale bars: 250 μm. The data underlying this figure can be found in S1 Data and S1–S3 Tables. PAG, periaqueductal gray; SC, superior colliculus; TPM, transcripts per million.

The deep SC/PAG highly expresses *Brn3b* but did not show obvious morphological alterations in *Brn3b* mutants. Considering the diverse roles of *Brn3b* in retinal development [21,30,31], we speculated that apart from specific structural changes, *Brn3b* loss could cause various transcriptional alterations, including dysregulation of the genes involved in visually triggered behavioral responses. To test this hypothesis, we conducted transcriptomic profiling of the dorsal midbrain and identified 1,992 genes that were significantly differentially expressed in *Brn3b* cKOs compared to controls (padj < 0.05; Fig 4A and 4B and S1–S3 Tables). We discovered that besides *Brn3b* itself, neuropeptide *tachykinin* 2 (*Tac2)* showed the highest level of down-regulation in the *Brn3b* mutants. Interestingly, *Tac2* was previously implicated in fear learning [32,33]. Additionally, *Tac2* expression was up-regulated by single housing-based social isolation, which caused enhancement of freezing responses to a visually threatening looming stimulus [34]. Therefore, we hypothesized that decreased *Tac2* expression due to the loss of *Brn3b* could affect neural circuits mediating fear-related behavior.

To further assess whether *Tac2* may act downstream of *Brn3b*, we performed reverse transcription-quantitative PCR (RT-qPCR) on independent biological samples and confirmed that *Brn3b* deletion indeed significantly reduces *Tac2* expression (approximately 50%, Fig 4C). Two related neuropeptides, *tachykinin1* (*Tac1*) and *corticotropin-releasing hormone* (*Crh*), known to mediate effects of stress [35,36], showed no difference between controls and mutants. To determine whether Brn3b+ neurons express *Tac2*, we performed in situ hybridization with *Tac2* probes on brain tissues of the *Brn3b*^GFP/+^ mouse (S10 Fig). Tac2+ cells were mainly located in the deep SC/PAG and approximately 57% of Tac2+ cells were Brn3b+. No Tac2+ cells were found in the superficial and intermediate SC. Furthermore, *Brn3b* deletion reduced Tac2+ cell number (approximately 37% decrease, Fig 4D–4G), consistent with the finding that *Brn3b* regulates *Tac2* expression. Unlike the superficial SC, altered *Tac2* expression in the deep SC/PAG of the mutants very unlikely resulted from neuronal death, as neither an obvious loss of AP signals (Fig 2A) nor a change in the number of Brn3a+ cells and GFP+ cells was detected in that area (S6 and S7 Figs).

### Brn3b-dependent circuits are required for behavioral responses to visual threat

**Fig 5:**
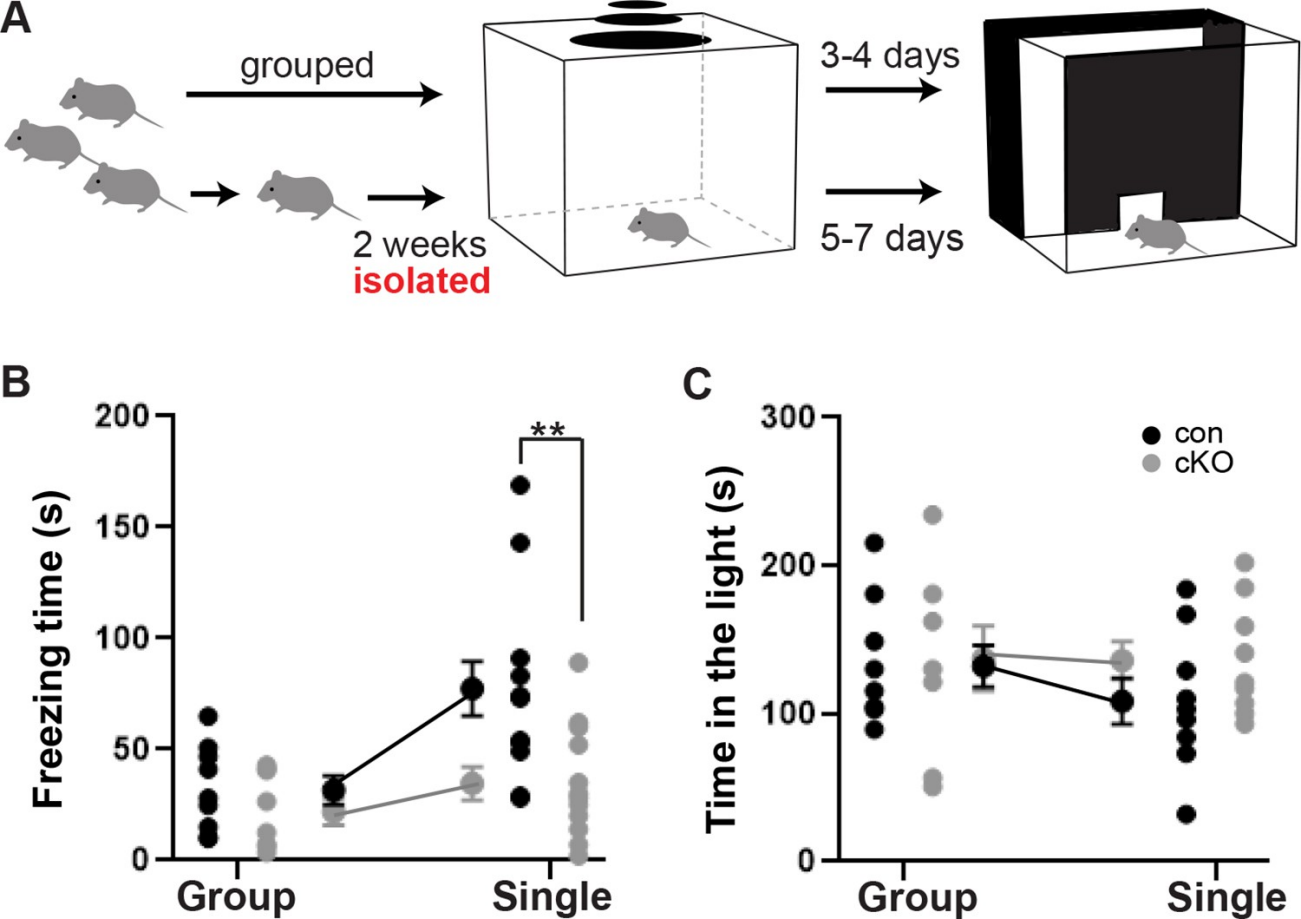
Brn3b-dependent circuits are necessary for visually triggered freezing responses. Fear-related defensive behavior was analyzed by examining freezing responses to visual threat in a looming stimulus assay. **(A)** Schematic diagram of the behavioral paradigms. The looming stimulus was an expanding dark disk on a gray background presented overhead (0.25 s expansion; 0.25 s at largest disk size; 0.5 s interstimulus interval; 10 repeats). Following the looming stimulus assay, the mice were tested on light/dark discrimination. For the experiments involving single housing, the mice were housed individually for 2 weeks prior to the behavioral tests. **(B)** Quantification of freezing responses for group-housed (32.4 ± 6.5 s for control, 22.6 ± 6.1 s for cKO; *n* = 9 for control and 8 for cKO) and single-housed (78.1 ± 12.3 s for control, 35.3 ± 7.4 s for cKO; *n* = 12 for control and cKO) mice. A significant difference in total freezing time was observed between control and cKO in single-housed but not in group-housed mice. Tukey post hoc analysis (mean ± SEM, *p* = 0.903 for group-housed and *p* = 0.006 [**] for single-housed animals). **(C)** Quantification of the time spent in the brightly lit area for group-housed (132.5 ± 14.0 s for control, 137.5 ± 21.9 s for cKO; *n* = 9 for control and 8 for cKO) and single-housed (108.7 ± 15.6 s for control, 136.1 ± 12.9 s for cKO; *n* = 9 for control and cKO) mice. No significant difference was detected in the light/dark exploration test. Tukey post hoc analysis (mean ± SEM, *p* = 0.996 for group-housed, *p* = 0.618 for single-housed animals). The data underlying this figure can be found in S1 Data.

Upon discovering that *Brn3b* loss disrupts SC-LP connections and down-regulates *Tac2* expression, we proceeded to evaluate whether *Brn3b* deletion affects visually triggered fear responses using the looming stimulus assay (Fig 5A). This assay examines the mouse’s reaction to a dark, overhead expanding disk (a looming stimulus) that mimics an approaching predator (see Methods) [18]. The stimulus causes mice to either freeze or escape to a shelter. Here, we omitted the shelter to focus on freezing responses that are thought to depend on the SC-LP pathway [9,10,14]. The behavioral examination was performed using group-housed as well as single-housed mice to investigate potential enhancement of the freezing responses precipitated by social isolation and linked to *Tac2* activity [34]. Basic visual abilities were examined using the light/dark exploration test [37,38].

Our looming stimulus assay evoked prolonged freezing responses (Fig 5B). Indeed, nearly all mice froze within a few seconds of the onset of the 10 s looming stimulus and remained frozen for up to 3 min in some cases. A 2 × 2 ANOVA (genotype × housing condition) showed a main effect of genotype (F = 7.866, *p* = 0.008) and a main effect of housing condition (F = 9.727, *p* = 0.004) with no interaction (F = 3.099, *p* = 0.087). Specifically, the *Brn3b* cKO group froze less than the control group, and both groups froze longer following single housing. Follow-up post hoc analysis showed a significant difference between groups only in the single housing condition (t = 5.018, *p* = 0.006; group housing: t = 0.965, *p* = 0.903). All mouse groups behaved similarly in the light/dark exploration test (Fig 5C). A 2 × 2 ANOVA showed no statistical differences between genotypes (F = 1.011, *p* = 0.323) and housing conditions (F = 0.6073; *p* = 0.442). Overall, these results suggest that *Brn3b* cKO mice have overall normal vision; however, they exhibit impaired visually evoked freezing responses following single housing.

### Tac2 overexpression in *Brn3b* mutants rescues freezing responses to visual threat

Our findings indicated that *Brn3b* deletion reduces *Tac2* expression in the deep SC/PAG and that *Brn3b* mutants exhibit decreased freezing reactions to visual threat precipitated by single housing. Given that single housing was reported to enhance freezing behavior via *Tac2* up-regulation [34], we speculated that decreased *Tac*2 expression in *Brn3b* mutants prevents visual fear augmentation induced by social isolation. However, due to a lack of direct evidence that increased expression of Tac2 in dorsal midbrain can affect visually triggered fear behavior, we first examined whether Tac2 up-regulation in the SC/PAG of WT mice is sufficient to enhance freezing responses to the looming stimulus (S11 Fig). To overexpress Tac2, we generated and delivered AAV-Tac2 as well as AAV-mCherry necessary for visualization. The fidelity of co-expressing 2 AAV constructs has been assessed previously [22] and *Tac2* overexpression was confirmed by RT-qPCR. AAV-mCherry alone was delivered as a control. Four to 6 weeks after AAV injection, behavioral experiments were conducted. Analysis revealed that Tac2 overexpression in the deep SC/PAG of WT mice is sufficient to increase freezing in the response to a visual threat but has no effect on general visual ability. Subsequent histological analysis verified expression of mCherry in the deep SC/PAG.

**Fig 6:**
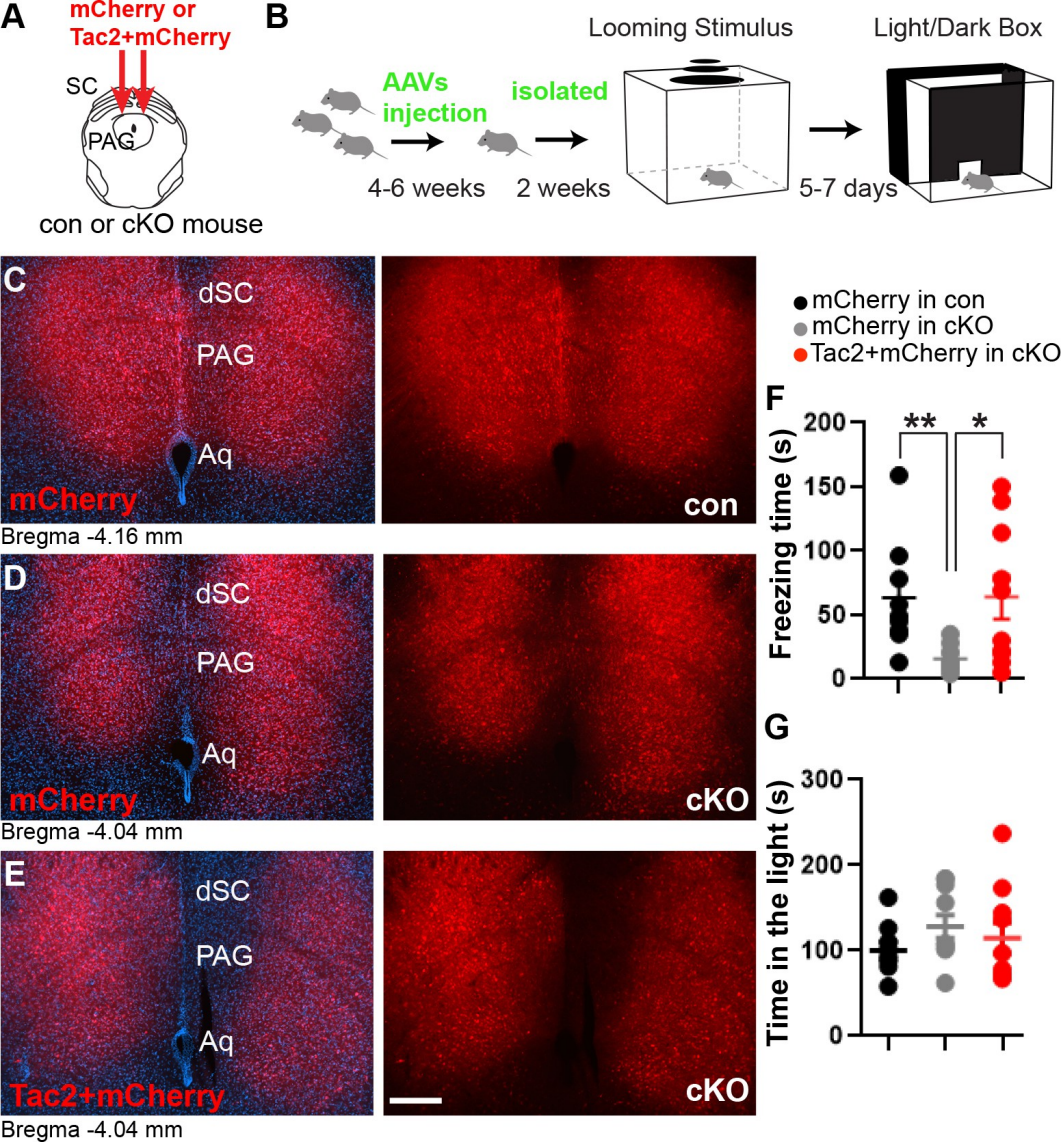
Overexpression of Tac2 ameliorates behavioral deficit in *Brn3b* mutants. **(A)** Schematic diagram of Tac2 + mCherry delivery into the deep SC/PAG layer. AAV was used as a delivery vehicle. **(B)** Schematic diagram of the behavioral assays. **(C–E)** Representative images of mCherry expression in control brains (red, **C**), mCherry expression in cKO brains (red, **D**), and Tac2 + mCherry co-expression in cKO brains (red, **E**). The sections were collected following behavioral assays. DAPI (blue). **(F, G)** Quantification of freezing responses in the looming stimulus assay (63.4 ± 14.4 s for mCherry in control, 15.9 ± 3.1 s for mCherry in cKO, 64.0 ± 17.2 s for Tac2 + mCherry in cKO; *n* = 9 for mCherry in control, 10 for mCherry and Tac2 + mCherry in cKO) and time spent in the brightly lit area in the light/dark exploration test (100.1 ± 10.1 s for mCherry in control, 128.2 ± 13.7 s for mCherry in cKO, 114.5 ± 18.1 s for Tac2; *n* = 9 for mCherry in control, 10 for mCherry and Tac2 + mCherry in cKO). Tac2 overexpression in cKO mice specifically increased their total freezing time, causing cKO animals to freeze similarly to control mice. No significant difference was detected during the light/dark exploration test. Unpaired two-tailed Student’s *t* test (mean ± SEM, *p* = 0.004 [**] or *p* = 0.01 [*] for freezing time; *p* = 0.123 or 0.553 for time spent in the light). Scale bars: 250 μm. The data underlying this figure can be found in S1 Data. AAV, adeno-associated virus; PAG, periaqueductal gray; SC, superior colliculus.

Next, we examined whether restoring *Tac2* levels in *Brn3b* mutants would ameliorate impairment in visually triggered defensive freezing (Fig 6A and 6B). We delivered AAV-Tac2 with AAV-mCherry into the deep SC/PAG of cKO mice. AAV-mCherry alone was injected into both control and cKO mice. Four to 6 weeks after AAV injection, animals were individually housed for 2 weeks prior to behavioral experiments.

The looming stimulus assay following the single housing showed that Tac2 overexpression rescued the behavioral deficit in the *Brn3b* mutants, increasing freezing responses to the level exhibited by control mice, thereby ameliorating behavioral deficit displayed in the mutants injected with AAV-mCherry alone (Fig 6F and 6G), whereas there was no effect in the light/dark exploration test. We also confirmed mCherry expression in the deep SC/PAG by the post hoc anatomical analysis (Fig 6C–6E). Collectively, our findings indicate that *Ta*c2 acts downstream of *Brn3b* in regulating visually triggered fear-related defensive behavior.

## Discussion

**Fig 7:**
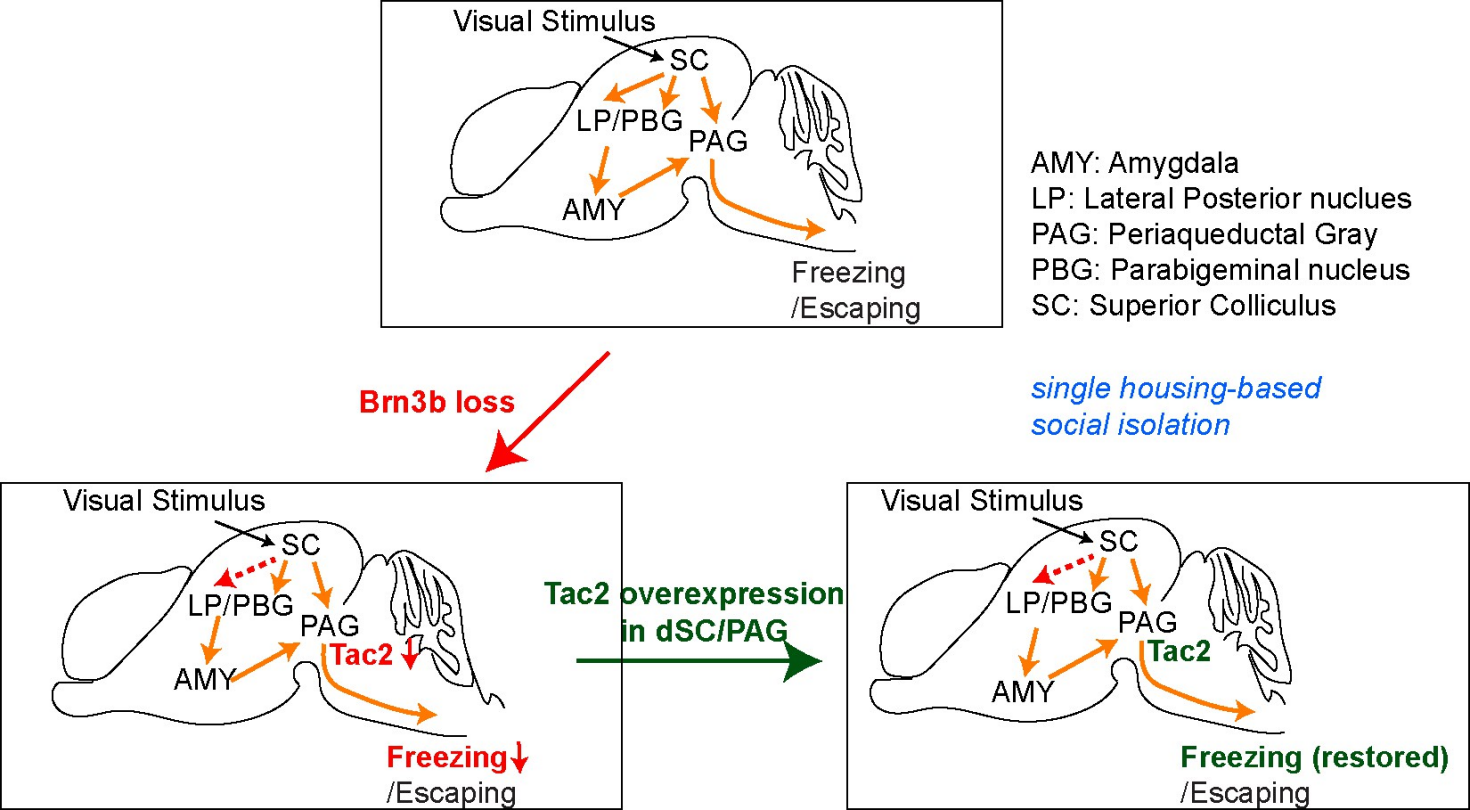
Schematic depiction of the circuits affected by *Brn3b* deletion and mediating Tac2 overexpression-dependent behavioral rescue.

Defensive responses to visually threatening stimuli represent an essential fear-related survival instinct. Diverse approaches, including optogenetic techniques, identified several distinct neural circuits associated with specific defensive responses to visual threat. However, we lacked a basic understanding of molecular mechanisms underlying organizational principles as well as functional development of those circuits. By combining molecular, genetic, anatomical, and behavioral analyses, we discovered a novel mechanism involved in development and function of midbrain fear-related visual circuitry. Specifically, we demonstrated that conditional deletion of *Brn3b* in the midbrain decreased axonal projections to LP due to neuronal loss in the superficial SC and also down-regulated *Tac2* expression in the deep SC/PAG. Behaviorally, *Brn3b* mutants showed diminished defensive fear responses to visual threat precipitated by single housing-based social isolation. Overexpression of *Tac2* in the deep SC/PAG of the mutants significantly increased their freezing responses, suggesting that while *Brn3b* appears to play a multifaceted role in regulating fear-related midbrain circuitry, *Tac2* functions as its important downstream effector in mediating visually triggered defensive behavior (Fig 7).

*Brn3b* controls development of RGCs and its deletion in the retina regulates RGC survival and axonal differentiation [16,17]. Also, retina-specific deletion of *Brn3b* was reported to affect visually triggered defensive responses [39], although it has been unclear whether behavioral changes were a direct consequence of RGC loss versus secondary effect related to disorganization of the superficial SC following the death of RGCs [17,26]. Moreover, we lack a basic understanding of the mechanisms of *Brn3b* function in the retina as well as the role of *Brn3b* in other parts of the nervous system. Here, we identified specific roles of *Brn3b* in the development and function of the midbrain sensory systems mediating visual fear responses. Although *Brn3b* deletion in the midbrain produces no obvious effects on axonal differentiation, this deletion strongly influences neuronal survival in the superficial SC. As our analysis of axonal development was performed en masse, it could have precluded identification of subtle anatomical changes. Although it would be difficult to detect such alterations by regular histological approaches, they (if present) could have contributed to our macroscopic level measurement of LP area. This could explain the discrepancy that only approximately 23% of LP projecting cells were Brn3b+ but there was approximately 48% decrease of LP area covered by AP signals in *Brn3b* mutants. Sparser labeling of axons coupled with deletion of *Brn3b* at a single-cell level may provide additional insights into *Brn3b* function.

*Brn3b* mutants exhibited both reduced superficial SC-LP projections and down-regulated expression of *Tac2* in the deep SC/PAG. What might be the relative contributions of these 2 phenotypes to the altered freezing responses of *Brn3b* mutants? Considering that *Brn3b* is expressed only in approximately 23% of Ntsr1+ neurons and that Tac2 overexpression in the deep SC/PAG of *Brn3b* mutants enhanced their freezing behavior following social isolation, *Tac2* seems to play a fundamental role as a critical downstream effector of *Brn3b* in regulating defensive fear responses. Nevertheless, the contribution of the diminished superficial SC-LP projections to freezing behavior cannot be completely ruled out because of the apparent importance of SC-LP projections to freezing behavior [9,10,14]. To evaluate the effect of the altered LP projections on behavioral changes in *Brn3b* mutants, we attempted to specifically delete *Brn3b* in the superficial SC using the *Ntsr1-GN209-Cre* line. However, this approach produced no obvious morphological changes. We speculate that Cre expression in the *Ntsr1-GN209-Cre* line may occur too late in development to cause significant anatomical alterations due to *Brn3b* loss. Further evaluation of the *Brn3b*-dependent phenotypes in the midbrain, including altered SC-LP projections and down-regulated *Tac2* expression, will require additional genetic tools that enable more specific manipulations within individual midbrain layers. Additionally, it would be interesting to define specific molecular and cellular mechanisms linking *Brn3b* loss to *Tac2* down-regulation.

Defensive fear responses to visual threat include both freezing and escaping reactions [18]. These 2 behaviors are apparently mediated by distinct midbrain circuitry [9,10,14]: freezing depends primarily on an SC-LP circuit, whereas escaping depends on an SC-parabigeminal nucleus (PBG) circuit. We found that Brn3b+ neurons project to the LP, but not to PBG, and while projections to LP were decreased in *Brn3b* mutants, the PBG innervation was intact. These findings suggest that *Brn3b* deletion is unlikely to have an obvious effect on escaping behavior. Indeed, an optogenetic activation of Tac2+ cells in the midbrain had no effect on the escaping responses [40]. Considering that freezing and escaping utilize different pathways, Tac2+ circuits seem to preferentially regulate freezing behavior. While the involvement of tachykinin in fear-related behavior appears to exist in different phyla, the exact behavioral outputs could vary between organisms. For example, activation of tachykinin-expressing neurons in the threatened *Drosophila* gates visual aversion by increasing locomotion, similar to escaping [41].

Additional projections from GABAergic neurons in the vLGN to SC are shown to regulate defensive fear responses to visual threat [42]. Brn3b mutants also exhibited decreased projections to vLGN, due to possible cell death in the intermediate SC (Figs 2 and S5). However, such changes were not quantified here because it is unclear what would be the functional significance of altered projections to vLGN and whether Brn3b+ axons innervate GABAergic neurons in the vLGN that control escaping responses to visual threat.

Single housing-based social isolation can influence multiple aspects of behavior [43–46], including responses to visually evoked fear (Fig 5; Zelikowsky and colleagues [34]). However, two-week-long social isolation was shown not to affect anxiety-related behavior of adult mice (Zelikowsky and colleagues [34]). Such social isolation paradigm also did not seem to alter anxiety-like behavior of control and *Brn3b* mutants in the light/dark exploration test (Fig 5). Therefore, it is very unlikely that anxiety was a significant factor in defensive behavior-related phenotypes observed in the looming stimulus assay following 2 weeks of single housing.

The social isolation-dependent increase in *Tac2* expression could impact neural circuits due to Tac2 binding to its receptor, NK3R, causing an increase in Ca^2+^ availability and leading to increased release of neurotransmitters and neuromodulators [34]. As a neuropeptide, Tac2 may have an endocrine signaling capability, acting both locally and at a distance. In the future, it would be informative to identify the sites where Tac2 acts, possibly through local delivery of the peptide. Additionally, it would be interesting to examine whether the looming stimulus increases Ca^2+^ in Tac2+ cells and whether Brn3b+ and Tac2+ neurons innervate specific subcortical areas that express NK3R and convey at least some of their behavioral effects via a Tac2/NK3R pathway at those sites.

As visual information is arguably among the major sensory stimuli capable of eliciting fear reactions in humans, mechanistic analysis of specific visual circuits mediating distinct fear responses should help us better understand and, potentially, develop more effective treatments for fear-related neuropsychiatric conditions, such as posttraumatic stress disorder.

## Materials and methods

### Ethics statement

All animal procedures were approved by the Institutional Animal Care and Use Committee at Yale University (protocol number 2022–11368) and were in compliance with federal guidelines (National Research Council (US) Committee).

### Animals

Both male and female animals were used in all studies. The age of the animals was specified in each experiment. In the experiments requiring timed pregnancy, the date of a copulation plug detection was considered to be E0.5.

The following mouse lines were used: (1) global *Brn3b* knockout [47]; (2) conditional *Brn3b* knockout [21]; (3) *Brn3b*^GFP/+^(this study); (4) *engrailed1-Cre* (*En1-Cre*, in which restricted Cre expression in the midbrain and hindbrain has been confirmed [22,23]); (5) *Ntsr1-GN209-Cre*, in which selective Cre expression was found in LP-projecting SC neurons [27,28]; (6) Ai14 (Cre-dependent td-Tomato expression; JAX # 007914); and (7) C57BL/6J (JAX # 000664).

### Mouse generation

The *Brn3b*^GFP/+^ mouse line was generated by the Yale Genome Editing Center using the CRISPR/Cas9 strategy. Briefly, 2 sgRNAs for *Brn3b* (for 5′ and 3′ ends) were designed using an online tool (http://crispor.tefor.net). A targeting vector, containing *GFP* with SV40 small t intron sequences flanked by homology arms to 162 bp upstream and to 100 bp downstream of *Brn3b* coding sequence, was synthesized by Genewiz. Two sgRNAs, the targeting vector and Cas9 were injected into fertilized zygotes that were then implanted in pseudo-pregnant females. PCR, followed by DNA sequencing analysis, identified 4 founders in which the *GFP* sequence replaced the open reading frame of *Brn3b*. All 4 founders were crossed to wild-type mice for 2 generations before conducting histological analysis. Double labeling showed approximately 95% overlap between GFP and Brn3b signals in the progeny of all 4 founders (1 animal/founder).

The sequences of the sgRNA were:

for 5′ end, GCTCCGGCCGGGTACTTCTC; for 3′ end, GGAGAAGGGTCCCTAAATGC.

The primer sequences for screening were:

for 5′ end, forward-GGGGACTATAACTCCACCGC, reverse-TCGATGCCCTTCAGCTCGAT;

for 3′ end, forward-AGGACGACGGCAACTACAGG, reverse-GCGAAACCGGTTCACAATCT.

The sequences of homology arms and small t intron were:

left arm: CGGAGCTAGCGGCCACACTGGGAGAACCGGGCCTGGAAGCAGTGGCGGCTGAGCACAACTTTGCAGTGTTGTTCCCTCTGCTGCTCCGGCCGGGTACTTCCTCAGAGGGTCGGGTAGCTGGGACCGGAGTGCGCCAGCGACGAGCGCGCCGCGCAAGGAAAG;

small t intron: GTAAATATAAAATTTTTAAGTGTATAATGTGTTAAACTACTGATTCTAATTGTTTGTGTATTTTAG;

right arm: GGACCCTTCTCCAGGGATGGCCCTTTCCCTTCGCCCTCTTTTTTTCTAACCCCCTTCTTGTCTCTTCTGCCTCTTTCCTTTCTACTTTGGCTATCAGAAA.

### Immunohistochemistry

Mice were anesthetized by intraperitoneal injection of 100 mg ketamine plus 10 mg xylazine/kg of bodyweight and perfused transcardially with 4% paraformaldehyde (PFA)/PBS. Following perfusion, the brains were dissected, postfixed overnight at 4°C, incubated successively with 15% sucrose/PBS and 30% sucrose/PBS overnight at 4°C, and sectioned with a cryostat (12 to 20 μm). For vibratome sectioning, the tissues were postfixed overnight at 4°C, washed with PBS, and sectioned (50 to 100 μm). For immunostaining, sections were washed twice with PBS, blocked with 3% donkey serum/0.1% Triton X-100/PBS for 30 min at room temperature, incubated with primary antibodies for 1 to 3 days at 4°C and then with secondary antibodies for 2 h at room temperature.

Primary antibodies used were: rabbit anti-GFP (1:1,000, Millipore, AB3080P), chicken anti-GFP (1:1,000, Aves Laboratories, GFP-1020), rabbit anti-cleaved caspase-3 (1:1,000, Cell Signaling Technology, 9661), rabbit anti-calbindin D-28K (1:4,000, Swant, CB38), rabbit anti-Ki67 (1:500, Thermo Scientific, RM9106), rabbit anti-ETV1 (1:1,000, Abcam, AB36788), rabbit anti-DsRed (1:1,000, Clontech, 632496), mouse anti-Brn3a (1:500, Millipore, MAB1585), goat anti-Brn3a (1:3,000, Santa Cruz, SC31984), mouse anti-calretinin (1:2,000, Millipore, MAB1568), goat anti-Brn3b (1:500, Abcam, AB235268), goat anti-Brn3b (1:250, Santa Cruz, SC31989). Secondary antibodies conjugated to Alexa Fluor-488, Cy3 or Alexa Fluor 647 were diluted at 1:500 (Jackson ImmunoResearch Laboratories, 703-545-155, 711-545-152, 711-165-152, 711-605-152, 715-165-151, 705-165-147, or Thermo Fisher, A-2202, A-11055).

For AP staining, brains were sectioned on a vibratome (50 μm for postnatal mice and 100 to 200 μm for embryos). The tissues were washed with 2 mM MgCl_2_/PBS twice, incubated for 2 h at 65°C to inactivate the endogenous AP activity and then washed 3 times with AP buffer (0.1 M Tris, 0.1 M NaCl, 0.05 M MgCl_2_ (pH9.5)). AP staining was conducted in the AP buffer with 2 mM levamisole and NBT/BCIP (50X, Roche, 11681451001) for 4 to 16 h. After staining was complete, the tissues were washed with PBS, postfixed with 4% PFA/PBS overnight and dehydrated through EtOH incubation (50%-75%-85%-95%-100%). Just before imaging, the tissues were cleared with benzyl benzoate and benzyl alcohol (2:1).

### Construction and generation of AAV

To generate an AAV vector carrying *Tac2*, a full-length *Tac2* was amplified from P1 mouse brain cDNA by generating BamHI and EcoRI restriction sites at each end and subcloned into the AAV vector (Addgene, #74291) [48]. Generation of AAV-YFP and AAV-mCherry constructs was previously described [22]. AAV-Cre construct was obtained from (Addgene, #55636) [49]. Detailed strategies of AAV-FLP-DOG and AAV-fDIO-mCherry were previously described [22,29].

AAV production was based on a triple-transfection, helper-free method, and AAVs were purified as described [22]. A plasmid carrying either AAV capsid 2/1 or 2/9 genes (UPenn Vector Core) was used. The titer of the purified AAVs was determined by quantitative PCR using primers that recognize WPRE; the concentrated titers were >10^13^ viral genome particles/ml in all preparations. Viral stocks were stored at −80°C.

### Transcriptome profiling (RNA-seq)

Dorsal midbrains were dissected at P2-P4, snap-frozen on dry ice and stored at −80°C. Frozen samples were sent to the Yale Center for Genome Analysis for RNA extraction, library preparation, and RNA sequencing. Briefly, total RNA was prepared using the RNeasy PowerLyzer Tissue & Cell kit (Qiagen). RNA quality was assessed by estimating the A260/A280 and A260/A230 ratios by nanodrop and Agilent Tapestation 4200 RNA Screen Tape Assay. Samples with RIN values of 7 or greater were used for library prep.

To generate cDNA libraries, 1 μg of total RNA per sample was enriched for polyA RNA and converted into libraries using the Kapa mRNA Hyper Prep Kit for Illumina Platforms (KAPA Biosystems). Briefly, mRNA was purified with oligo-dT beads and sheared by incubation at 94°C in the presence of Mg. Following first-strand synthesis with random primers, second strand synthesis and A-tailing were performed with dUTP for generating strand-specific sequencing libraries. Adapter with 3′ dTMP overhangs were ligated to library insert fragments. Library amplification amplified fragments carrying the appropriate adapter sequences at both ends. Strands marked with dUTP were not amplified. Indexed libraries that meet appropriate cut-offs for both were quantified by qRT-PCR using a commercially available kit and insert size distribution was determined by the LabChip GX or Agilent Bioanalyzer. Samples with a yield of ≥0.5 ng/μl were used for sequencing. The samples were sequenced on an Illumina NovaSeq S2. Briefly, the sample concentrations were normalized to 1.2 nM and loaded onto an Illumina NovaSeq flow cell at a concentration that yields 25 million passing filter clusters per sample. Samples were sequenced using 100 bp paired-end sequencing on an Illumina NovaSeq according to Illumina protocols.

Low-quality base calls, sequences with low-complexity tails and adaptor sequences were removed using fqTrim2.py (Yale Center for Genome Analysis). Trimmed paired-end (PE) reads were aligned to the mouse genome (GRCm38.p5) using Tophat2/Bowtie 2 [50]. Raw read counts per gene were calculated using HTSeq (version v.0.6.1p1) and expression levels in transcripts per million (TPM) were estimated by StringTie (version 1.3.3b) on GENCODE vM15 annotation [51,52]. Three independent replicates for control and 4 for *Brn3b* mutants were used for further analysis. Differential expression analysis was performed using DESeq2, which produced fold changes and statistical significance of changes between samples [53].

### Reverse transcription-quantitative PCR (RT-qPCR)

Midbrains including SC and PAG (around central aqueduct) were isolated. Total RNA was prepared using the RNeasy Mini Kit (Qiagen) and cDNA was synthesized using the Superscript III First-Stand Synthesis SuperMix (Thermo Fisher, #18080–400). RT-qPCR was performed in duplicates using iQ SYBR Green Supermix (Bio-Rad) on the CFX96 real-time system (Bio-Rad). The Ct values of the samples were normalized to that of *GAPDH* and expression level of each gene relative to that of controls was calculated by ΔΔCt method. Primers used for qPCR were as follows: *GAPDH*, 5′-GTGGAGTCATACTGGAACATGTAG-3′ and 5′-AATGGTGAA GGTCGGTGTG-3′; *Tac1*, 5′-TTTCGTAGTTCTGCATCGCG-3′ and 5′-TGGCCAGATCTCTCAC AAAA-3′; *Tac2*, 5′- GATGTCTCCTTTGGTCCCAC-3′ and 5′-AGGGAGGGAGGCTCAGTAAG-3′; *Crh*, 5′-ATCTCTCTGGATCTCACCTTCC-3′ and 5′-CCCGATAATCTCCATCAGTTTCC-3′; *Brn3b*, 5′-ATTGAAGAGCTCCGGCTTAG-3′ and 5′-CTGTCACACAACAACATGATCG-3′.

### In situ hybridization

*Tac2*, *Slc17a6*, and *Gad1* expression in brain tissues was detected by in situ hybridization following the method previously described [15]. Briefly, we amplified an entire coding region of *Tac2* (NCBI reference sequence, NM_009312.2) and *Slc17a6* (NM_080853) or the partial coding sequence of *Gad1* (NM_008077) from the brain cDNA and subcloned into pCR8/GW/TOPO TA vector (Invitrogen) or pGEM Teasy (Promega). Antisense riboprobes were synthesized using digoxigenin-labeled UTP. Signals were detected by peroxidase-conjugated anti-digoxigenin antibodies and by the tyramide signal amplification system (TSA-Plus, PerkinElmer Life Sciences). To examine overlap between Tac2+ and Brn3b+ cells in *Brn3b*^*GFP/+*^ mouse brain, the signals were first amplified by anti-digoxigenin antibody to Tac2 and then by antibodies to GFP (i.e., Brn3b).

### Intraocular injection and enucleation

Animals received analgesic buprenorphine (0.05 to 0.1 mg/kg of body weight) before surgery and were then anesthetized with a mixture of 100 mg ketamine plus 10 mg xylazine/kg of bodyweight. All drugs were administrated intraperitoneally. To visualize axonal distribution of RGCs in the SC, a small hole was made in an eye with an insect pin (size 00) to release intraocular pressure. Cholera toxin B subunit conjugated to Alexa Fluor-555 (CTB-555; 1 μl of 1 mg/ml, Invitrogen) was injected through the same hole using a Hamilton syringe. Intraperitoneal injections of buprenorphine (0.05 to 0.1 mg/kg of body weight) were given for 48 h after the surgery.

For enucleation, the mice were treated with buprenorphine and meloxicam and anesthetized with a mixture of ketamine and xylazine described above. Before removal of eyes, lidocaine (<7 mg/kg of body weight) was administrated around the surgical area by subcutaneous injection. The eyes were removed using sterile surgical scissors. Eyelids were sutured to prevent unwanted infection using surgical specialties. Animals then received intraperitoneal injection of buprenorphine (0.05 to 0.1 mg/kg of body weight) and meloxicam (1 to 5 mg/kg of body weight) for 48 h after surgery.

### Brain injections

To trace axonal projections from distinct layers of the midbrain, we injected AAV-mCherry (or AAV-YFP) into wild-type mice or AAV-Cre into *Brn3b*^flox/+^ mice. The mice received intraperitoneal injection of buprenorphine (0.05 to 0.1 mg/kg of body weight) before surgery and were then anesthetized by intraperitoneal injection of a mixture of 100 mg ketamine plus 10 mg xylazine/kg of bodyweight before being mounted on a stereotaxic apparatus. Lidocaine (<7 mg/kg of body weight) was administrated around the surgical area by subcutaneous injection before incision. A small craniotomy was made over the SC using a dental drill. Coordinates used for injection were bregma −4.5 mm, lateral ± 0.6 mm, dura −1.2 mm (for the superficial SC), and −2.4 mm (for the deep SC/PAG). AAVs (approximately 50 nl) were unilaterally injected with a glass pipette at a rate of approximately 10 nl/min, and the pipette was left in place for 5 min after injection. Enucleation was conducted after the brain injection on the same day when AAVs were delivered into the intermediate SC. Intraperitoneal administration of buprenorphine (0.05 to 0.1 mg/kg of body weight) and meloxicam (1 to 5 mg/kg of body weight) was given for 48 h after the surgery. Three to 4 weeks later, the brain tissues were dissected and processed to visualize axonal projections.

To overexpress Tac2, we injected a mixture of AAV-Tac2 and AAV-mCherry, or AAV-mCherry alone for control into the deep SC and PAG. General surgical procedures were performed as described above. Coordinates used for injection were bregma −4.5 mm, lateral ± 0.6 mm, dura −2.4 mm. Four to 6 weeks later, the animals were single-housed for 2 weeks before conducting behavioral tests. After completion of the tests, the brain tissues from all tested animals were dissected, sectioned, and imaged to confirm mCherry expression in the deep SC and PAG.

To label superficial Brn3b+ neurons by FLP-DOG strategy, we injected AAV-FLP-DOG and AAV-fDIO-mCherry into the SC of *Brn3b*^GFP/+^ mouse at P1. Mice received oral injection of meloxicam (0.3 mg/kg of bodyweight) before surgery and were anesthetized by chilling on ice. Pups had no direct contact with ice, as they were placed on a barrier covering the crushed ice. A small incision (maximum length 3 mm) was made on the skin over the SC. The skull and brain tissue were penetrated at the same time by disposable glass pipettes (<0.1 mm outer diameter) containing AAVs. A pressure injector was used to deliver approximately 50 nl of AAVs unilaterally. The pipette was gently withdrawn after injection and the skin was sealed using cyanoacrylate glue. Oral injection of meloxicam was given for 48 h after surgery. Three to 4 weeks later, the brain tissues were dissected and further processed for histological analysis.

### Behavioral tests

Both male and female mice (16 to 20 weeks old) were utilized in the behavioral experiments; the subject numbers were sex-adjusted. Different behavioral assays were conducted 3 to 4 days apart for group-housed mice and 5 to 7 days apart for single-housed mice to allow for additional post-experimental recovery of the animals that experienced social isolation. All behavioral tests were performed and analyzed in a double-blind fashion.

Visually triggered defensive behavior was tested using a looming stimulus assay as described in Yilmaz and Meister. Briefly, the behavioral apparatus consisted of an open field-type arena with a computer monitor mounted on the top of the plexiglass chamber. All sidewalls of the chamber (40 × 40 × 30 cm) were coated with a matte finish (Krylon) to create a unidirectional visual perspective. The mouse was initially habituated to the test room for approximately 45 min and then placed into the arena to acclimate for approximately 10 min. During this time, the computer monitor illuminated the arena with a gray screen (mean luminance). Mouse behavior in the arena was recorded by a camera mounted on the chamber and the video was stored on a computer using video capture software. Following acclimation, the computer screen, regulating illumination of the test area, began presenting an expanding dark spot—a negatively contrasted circle expanding from 2 to 20 degrees of visual angle over 250 ms and then remaining at its largest size for an additional 250 ms. After a 500-ms interstimulus interval, during which the gray screen was presented, the looming stimulus repeated for a total of 10 times (1 stimulus s^-1^). The stimulus was generated in MATLAB. Mouse freezing responses were tested in the absence of a shelter and analyzed using Boris 7.13, an open-source event tagging software [54]. Freezing time was calculated as a time period when the mouse remained completely immobile during 3 min of poststimulus video recording.

Visual discrimination ability was tested using a light/dark exploration test as previously described [38,55]. Briefly, the light/dark chamber (40 × 40 × 30 cm) was made of transparent plexiglass and contained a black plexiglass exploration box (20 × 40 × 30 cm) occupying about half of the total chamber area. The dark box had a removable black plexiglass cover and small exit/entrance space at the center. The light compartment was diffusely illuminated by a bright 120 W lamp centered over the lit area. A mouse was habituated to the test room for approximately 45 min before the experiment. A trial began by placing the mouse into the dark compartment and allowing it to freely explore the chamber. Mouse behavior was video recorded for 10 min. The video-recorded behavior, including the total time spent by the animal in the brightly lit area, was analyzed manually using Boris version 7.13.

### Image acquisition and analysis

Fluorescent images were collected using a Zeiss Imager M2 fluorescence microscope and a Zeiss LSM 800 confocal microscope. The *z*-stacks were obtained with 1 μm steps using a 20× objective (NA 0.8) or 4 μm steps using a 10× objective (NA 0.45). AP images were acquired using a Zeiss Axioskop equipped with SPOT software (Diagnostic Instruments, Sterling Heights, Michigan, United States of America). Data analysis was conducted by ImageJ software (National Institutes of Health, Bethesda, Maryland, USA).

To indicate anatomical location of brain sections/images used for analysis, the Bregma levels were indicated on the figures depicting sections obtained from approximately 24-day-old mice. For younger animals when the brain is still developing and/or a clear Bregma is lacking, the following localization and visualization approaches were employed. For coronal images used for analysis, diagrams of the coronal sections showed their specific brain positions and the corresponding sagittal images depicted the levels where each section was obtained. Anatomical structures of the coronal sections were defined using information from the Atlas of the Developing Mouse Brain [56]. For sagittal images used for analysis, the level of each section was shown as a lateral distance from the midsagittal line. For S8 Fig, the anatomical level for images was described in the figure legend.

To quantify axonal projections to the LP, AP images were taken using 50-μm thick coronal sections, approximately 300 μm apart, extending from the suprachiasmatic nucleus to the rostral cerebellum. Sections containing the LP adjacent to the dorsal lateral geniculate nucleus (dLGN) were chosen for further analysis to ensure the correct LP location. The poor quality of antibodies to AP made it difficult to conduct double immunostaining with an antibody to calretinin, which demarcate LP [22]. Therefore, LP localization was determined based on anatomical criteria. The areas covered by axon terminals were measured using ImageJ. Total LP areas from 2 or 3 sections (depending on the animal’s age) were combined, quantitatively compared to controls, and presented as percentages indicating relative differences. This approach allowed us to rule out the possibility that different sectioning levels along the anterior-posterior axis were compared between animals. To visualize dLGN and LP by double immunostaining at P10, antibodies to calbindin-D28K were used [57].

To assess and compare the total number and distribution of cells stained with specific antibodies, sagittal sections anatomically matched in controls and mutants, were used unless noted otherwise. Images were taken from the sections located at approximately 100 μm from the medial edge and continued laterally, approximately 50 μm apart by E16, and approximately 75 μm apart by P1/P2. For cell counting, the images were cropped to 350× up to 450 μm at E15; to 350× up to 500 μm at E16; to 350× up to 800 μm at E18; and to 350 × 1,000 μm at P1/P2. Such cropping covers an area from the pia to the dorsal PAG at each developmental stage. For ETV1+ neurons, the entire image was used for the analysis. To assess the distribution of Brn3a+ neurons and GFP+ neurons, the counted cells were grouped within 50 μm intervals for E15/E16 analysis and within 100 μm intervals for P1 analysis. To analyze the number of Tac2+ neurons, the images were taken from coronal sections located just rostrally to the inferior colliculus and continued rostrally approximately 240 μm apart at P13-P18; the images were cropped to 700 × 700 μm for counting.

### Experimental design and statistical analysis

Analysis of axonal projections was conducted in 2 to 3 different sections per animal and averaged for 4 to 5 animals per condition. The number and distribution of cells were analyzed using 2 to 3 different sections per animal and averaged for 3 to 5 animals per condition. Animals of either sex were utilized for the analysis.

All data were reported as mean ± SEM and analyzed using GraphPad Prism software (GraphPad Software; Dotmatics, San Diego, California, USA). Statistical analyses were conducted using an unpaired two-tailed Student’s *t* test when comparing means between 2 groups or two-way ANOVA followed by Tukey post hoc test when comparing means between multiple groups. The number of animals used per condition, the mean values ± SEM for each data set, and statistical significance were provided in each figure/figure legend. Statistical significance was determined when *p* < 0.05 (*), *p* < 0.01 (**), *p* < 0.001 (***), *p* < 0.0001 (****).

## Supporting information

S1 Fig Brn3b expression in the dorsal midbrain, thalamus, and retina. (**A–C**) Schematic diagrams of coronal images showing the brain area (boxed) used for analysis (left) and sagittal images depicting the level (dashed line) where the coronal section was obtained (right) at P1 (**A**) and P9-P12 (**B** and **C**). (**D–G**) Brn3b expression in *Brn3b*^flox/+^:: *En1-Cre* mouse, visualized by immunostaining at P1 (**D**) and P10 (**F**) and by AP labeling at P1 (**E**) and P12 (**G**). A magnified view of the boxed areas (**i–iv**) showed that Brn3b+ neurons are located close to the pia at P1 and become confined to the SO layer at P10/P12 (*n* = 3 mice/age/visualization method). Due to the poor quality of AP antibody staining and the required tissue clearing for AP staining, immunostaining and AP labeling were conducted using different sections/animals. (**H**, **I**) Brn3b immunostaining in WT mouse at P9. Brn3b expression is missing in the upper layer of superficial SC (SGS: **H**), consistent with observation in the *Brn3b*^flox/+^:: *En1-Cre* mice. No Brn3b signals were detected in LP (**I**). Immunostaining with anti-Brn3b was conducted at P9 because of very weak signals produced by Brn3b antibody beyond P12 (*n* = 3 mice). To demarcate dorsal lateral geniculate nucleus (dLGN) and LP in the thalamus, calbindin antibody (green) was used (Grubb and colleagues [57]). (**J**) No AP signals were detected in the retina of *Brn3b*^flox/+^:: *En1-Cre* mouse (*n* = 3 animals). Dashed lines indicate either the pia surface or boundary of dLGN and LP (**D–I**). Blue (DAPI). Scale bar: 200 μm.
(TIF)

S2 Fig Brn3b+ neurons in the dorsal midbrain of *Brn3b*^GFP/+^ mice are glutamatergic. (**A**) CRISPR/Cas9-mediated genome-editing strategy to generate *Brn3b*^*GFP/+*^ (*Brn3b-GFP*) knock-in mouse line. Two sgRNAs (for 5′ and 3′ ends) were utilized. The targeting vector contains the *GFP* with a small t intron flanked by homology arms (indicated by gray boxes) to 162 bp upstream and 100 bp downstream of *Brn3b* open reading frame. This strategy produces a mouse in which the *GFP* sequence replaces an open reading frame of *Brn3b*. (**B**) Schematic diagrams of a coronal image showing the brain area (boxed) used for analysis (top) and a sagittal image depicting the level (dashed line) where the coronal section was obtained (bottom). (**C**) (Left) A section of *Brn3b*^*GFP/+*^ mouse brain labeled with antibodies to GFP (green) and Brn3b (red) at P10. (Right) Magnified view of the boxed areas (**i, ii**). Quantification revealed that approximately 95% of Brn3b+ cells express GFP (1,178.8 ± 41.6 cells/animal, *n* = 4 animals) and approximately 93% of GFP+ cells express Brn3b (1,189.0 ± 43.1 cells/animal, *n* = 4 animals), confirming that GFP+ cells faithfully represent Brn3b+ neurons. Orange arrows indicate examples of overlapping signals. DAPI (blue). (**D**) Schematic diagram showing the brain areas of the *Brn3b*^*GFP/+*^ mouse analyzed using double labeling by in situ hybridization with probes to *Slc17a6* and *Gad1* and immunostaining with anti-GFP antibody. (**E–L**) Brn3b+ neurons (green, visualized by GFP antibody) express *Slc17a6* (red, **G–I**) but not *Gad1* (red, **J–L**). Quantification of overlap between *Slc17a6* and GFP (**E**: 834.7 ± 47.7 cells/animal, *n* = 3 animals) or between *Gad1* and GFP (**F**: 861.7 ± 17.9 cells/animal, *n* = 3 animals). Orange arrows indicate examples of overlapping signals; white arrows indicate examples of non-overlapping signals. Scale bars: 250 μm (**C**), 50 μm (**G–L**). The data underlying this figure can be found in S1 Data.
(TIF)

S3 Fig Loss of Brn3b expression in the dorsal midbrain of conditional *Brn3b* mutants. (**A**) Schematic diagrams showing the brain area (boxed) of a coronal section used for analysis (top) and a sagittal diagram depicting the level (dashed line) where such coronal section was obtained (bottom) at P1 (**i**) and at P10-P13 (**ii**). Brn3b expression (red) in control (**B** and **D**) and cKO (**C and E**) brains, visualized by immunostaining (*n* = 3 mice/group/development stage). DAPI (blue). Scale bars: 200 μm.
(TIF)

S4 Fig Projections of Brn3b+ neurons in superficial SC to other subcortical areas. (**A–D**) *Ntsr1-GN209-Cre* line expresses Cre in the superficial SC neurons projecting to LP (Gale and Murphy [27]). (**A**) The retina of *Ntsr1-GN209-Cre*:: *Brn3b*^*flox/+*^ mouse showing AP signals. (**B**) Brn3b+ neurons in superficial SC (left, arrow) and projections to LP (right), visualized by AP signals in control brain (*Ntsr1-GN209-Cre*:: *Brn3b*^*flox/+*^). (**C**) No structural changes were detected in the mutants (*Ntsr1-GN209-Cre*:: *Brn3b*^*flox/-*^). To ensure that the labeled axons in the LP originated from the SC, and not the retina, both eyes were enucleated. Additional AP covered areas were detected in the midbrain, suggesting that Cre expression pattern in the *Ntsr1-GN209-Cre* line differs from the pattern in *En1-Cre* (*n* = 3 mice). (**D**) (Left) Schematic diagram showing the brain area analyzed to examine Brn3b expression in *Ntsr1-GN209-Cre*:: Ai14 mice expressing Cre-dependent td-Tomato. (Right) Overlap between Brn3b (green) and td-Tomato (red). Quantification revealed that approximately 23% of td-Tomato+ cells were Brn3b+ (604.3 ± 41.3 cells/animal, *n* = 3 animals). Arrows indicate examples of overlapping signals. (**E**) Schematic diagram depicting the FLP-DOG/ fDIO-mCherry strategy. FLP-DOG is unstable and degrades in the absence of GFP. Binding of GFP stabilizes FLP-DOG, which coverts fDIO-mCherry to an active form resulting in mCherry expression (Flpo, codon-optimized FLP; dGBP1, destabilized GFP-binding protein; Ub, ubiquitin molecules; CAG, promoter). (**F**) Schematic diagram of AAV-FLP-DOG and AAV-fDIO-mCherry delivery into the SC of *Brn3b*^GFP/+^ mouse. (**G**) Representative image of mCherry expression in the SC. Magnified view of the boxed areas showing overlap between mCherry+ and GFP+ (i.e., Brn3b+ cells) in superficial SC (**i**) but no mCherry signals in deep SC/PAG (**ii**). Arrows indicate examples of overlapping signals. (**H**) Quantification revealed that approximately 98% of mCherry+ cells express GFP (947 cells, *n* = 4 animals). (**I**) Projections of mCherry+ axons to LP. Dense GFP labeling in dLGN and vLGN indicates RGC axonal innervation. Scale bars: 500 μm (**A–C, G,** and **I**) and 50 μm (**D**). The data underlying this figure can be found in S1 Data.
(TIF)

S5 Fig Projections of Brn3b+ neurons in the intermediate or deep SC/PAG to other subcortical areas. (**A**) Schematic diagram of AAV-mCherry injection into deep SC/PAG. (**B**–**D**) Representative images showing mCherry expression in deep SC/PAG (**B**) and mCherry+ neuronal projections to LP (arrow in **C**). Magnified view of the boxed area (**D**). LP was identified by immunostaining with calretinin (CR) antibody (Byun and colleagues [22]) (*n* = 3 mice; DAPI (blue)). (**E**) Schematic diagram of AAV-Cre injection into intermediate SC of *Brn3b*^*flox/+*^ mouse. (**F, G**) Representative images of AP signals in intermediate SC (**E**) and projections to ventral LGN (arrow in **G**), indicating that Brn3b+ neurons in the intermediate SC project to ventral LGN (*n* = 2 mice). Very faint signals in LP (**G**) might originate from neurons in other layers of the dorsal midbrain. However, if other layers beyond the targeted areas were obviously labeled by the AAV, such animals were excluded from analysis (**A–G**). Scale bars: 500 μm.
(TIF)

S6 Fig Developmental analysis of Brn3b+ neurons in the dorsal midbrain using additional markers. (**A, B**) Schematic diagram illustrating the level of the sagittal sections as a lateral distance from the midsagittal line at E15, E16, P1, and P2 (**A**) and the layer distribution of Brn3b+ versus ETV1+ neurons and Brn3b+ versus Brn3a+ neurons in the dorsal midbrain (**B**). (**C–F**) Sections stained for Brn3b (red) and ETV1 (green) at E16 (**C, D**) and P2 (**E, F**). Neither Brn3b expression nor ETV1+/ Brn3b+ neurons in superficial SC were detected at E16 in control (**C**). Segregation of Brn3b+ neurons into superficial SC and intermediate SC was detectable at P2 in control (**E**). Some Brn3b+ neurons in the intermediate SC but not in superficial SC express ETV1 (arrows). (**G**) Quantification of ETV1+ neurons shows no clear difference between control and cKO (218.5 ± 15.4/ mm^2^ for control, 193.8 ± 17.8/ mm^2^ for cKO at E16, 263.8 ± 14.4/mm^2^ for control, 244.3 ± 12.4/ mm^2^ for cKO at P2; *n* = 4 mice/group/developmental stage). Unpaired two-tailed Student’s *t* test (mean ± SEM; *p* = 0.333 at E16; *p* = 0.345 at P2). (**H–K**) Sections stained for Brn3b (red) and Brn3a (green) at E15 (**H, I**) and P1 (**J, K**). No double Brn3b/Brn3a staining was conducted at E15 due to incompatibility of Brn3b and Brn3a antibodies (both raised in goat). Mouse antibody to Brn3a, used at P1, did not produce any signals at E15, likely due to a low level of Brn3a at this stage. (**L, M**) Quantification of Brn3a+ neuronal number and position at E15 and P1 showed no obvious difference between control and cKO (4,641.9 ± 96.2/mm^2^ for control, 4,656.7 ± 421.4/mm^2^ for cKO at E15, 1,985.5 ± 85.4/mm^2^ for control, 1,898.5 ± 118.2/mm^2^ for cKO at P1; *n* = 3 mice/group/developmental stage). Unpaired two-tailed Student’s *t* test (mean ± SEM; *p* = 0.974 at E15; *p* = 0.583 at P1). The dashed lines delineate the pial surface (**C–F** and **H–K**). DAPI (blue). Scale bars: 50 μm. The data underlying this figure can be found in S1 Data.
(TIF)

S7 Fig Developmental analysis of Brn3b+ neurons in the dorsal midbrain using the *Brn3b*^GFP/+^ mouse. (**A**) Schematic diagram of a coronal image showing the brain areas (boxed) used for analysis (left) and a sagittal image depicting the level (dashed line) where a coronal section was obtained (right) at P9/P10. (**B**) Schematic diagram illustrating the level of the sagittal sections as lateral distances from the midsagittal line at E16 and P1/2. (**C, D**) Double immunostaining with anti-Brn3b (red) and anti-GFP (green) shows no Brn3b expression in the *Brn3b*^GFP/flox^:: *En1-Cre* at P9/P10. Magnified view of the boxed areas showing the loss of GFP+ (i.e., Brn3b+) neurons in the superficial SC (**i, ii**), indicated by arrows (*n* = 3 animals/genotype). DAPI (blue). (**E, F**) Representative images of sections stained for Brn3b (red) and GFP (green) at E16. DAPI (blue). (**G**) Quantification of GFP+ neurons (**iii**) and their distribution (**iv**) showed no obvious differences at E16 (2,306.9 ± 83.7/mm^2^ for *Brn3b* ^GFP/+^, 2,319.6 ± 56.6/mm^2^ for *Brn3b* ^GFP/flox^:: *En1-Cre*, *n* = 3 animals/genotype). Unpaired two-tailed Student’s *t* test (mean ± SEM; *p* = 0. 906). (**H, I**) Representative images of sections stained for Brn3b (red) and GFP (green) at P1/2. DAPI (blue). (**J**) Quantification of GFP+ neurons (**v**) and their distribution (**vi**) revealed that Brn3b loss decreases the number of neurons in the superficial and intermediate layers (613.6 ± 37.8/mm^2^ for Brn3b ^GFP/+^, 431.3 ± 39.6/mm^2^ for *Brn3b* ^GFP/flox^:: *En1-Cre*, *n* = 3 animals/genotype). Superficial and intermediate layers were defined as located within 350 μm from the pia. Unpaired two-tailed Student’s *t* test (mean ± SEM; *p* = 0.029 [*] for the cell number and *p* = 0.013 [*] for the distribution). Magnified view of the boxed area (**vii**). No clear difference was detected in the deep SC/PAG (1,857.1 ± 65.4/mm^2^ for *Brn3b* ^GFP/+^, 1,863.0 ± 101.0/mm^2^ for *Brn3b* ^GFP/flox^:: En1-Cre, *n* = 3 animals/genotype). Unpaired two-tailed Student’s *t* test (mean ± SEM; *p* = 0. 964). The dashed lines delineate the pial surface (**C–F** and **H–I**). DAPI (blue). Scale bars: 200 μm (**C–D**) and 50 μm (**E–F** and **H–I**). The data underlying this figure can be found in S1 Data.
(TIF)

S8 Fig Brn3b+ neuronal projections during embryonic development. Neuronal projections were visualized by AP signals at E13 (**A, B**), E16 (**C, D**), and P1/P2 (**E, F**). Projections to the ventral midbrain, including tegmentum, pons, and medulla, were detected during embryonic development and disappeared after birth. Overall, no differences were found between control and cKO (*n* = 3 mice/group/developmental stage). The images were taken using consecutive sections covering the entire superior colliculus, 200 μm apart at E13, 200 μm apart at E16, and 600 μm apart at P1/P2. Scale bars: 500 μm.
(TIF)

S9 Fig Decreased projections to LP in *Brn3b* mutants occur postnatally. Axonal projections were visualized by AP signals. (**A**) Schematic diagram of a coronal image showing the brain area (boxed) used for analysis (top) and sagittal images depicting the level (dashed line) where each coronal section was obtained (bottom) at P1-P5 and P8-P13. (**B**) (Left) Axonal branches in LP were barely detectable at P1/P2 in control and cKO mice (*n* = 4 mice/group). (Right) Magnified view of the boxed areas. (**C**) (Left) Axonal branches in LP were clearly noticeable at P4/P5 in control but barely visible in cKO mice (*n* = 4 mice/group). (Right) Magnified view of the boxed areas showing the LP regions used for analysis. (**D**) Quantification was presented as a relative difference between the AP-covered LP area in controls and mutants (1.00 ± 0.07 for control, 0.20 ± 0.03 for cKO; *n* = 4 mice/group). (**E**) (Left) Axonal branches in LP became abundant at P8/P9 in control and cKO mice, but the LP area covered by AP signals was smaller in cKO (*n* = 4 mice/group). (Right) Magnified view of the boxed areas. (**F**) Quantification (1.00 ± 0.07 for control, 0.40 ± 0.04 for cKO). (**G**) (Left) Axonal innervation in the LP is complete at P12/P13 in control mice, however, clearly decreased in cKO animals (*n* = 4 mice/group). (Right) Magnified view of the boxed areas. (**H**) Quantification (1.00 ± 0.09 for control, 0.45 ± 0.03 for cKO); unpaired two-tailed Student’s *t* test (mean ± SEM; *p* < 0.0001 [****] at P4/P5; *p* = 0.0004 [***] at P8/P9; *p* = 0.0012 [**] at P12/P13). Scale bars: 250 μm. The data underlying this figure can be found in S1 Data.
(TIF)

S10 Fig *Tac2* expression in Brn3b+ neurons in the *Brn3b*^GFP/+^ mouse. (**A**) Schematic diagrams of a coronal image showing the brain areas (boxed) used for analysis (left) and a sagittal image depicting the level (dashed line) where each coronal section was obtained (right). (**B, C**) Brain sections of *Brn3b*^*GFP/+*^ mouse labeled with anti-GFP (i.e., Brn3b; green) and in situ probe to *Tac2* (red) at P18. (**B**) (Left) No *Tac2*+ signals were detected in the superficial and intermediate SC layers. (Right) Magnified view of the boxed areas (**i, ii**). (**C**) (Left) *Tac2*+ cells are detectable in deep SC/PAG. (Right) Magnified view of the boxed areas (**iii, iv**). Quantification revealed that approximately 57% of Tac2+ cells are Brn3b+ (70.7 ± 7.2 cells/animal, *n* = 3 animals). Arrows indicate examples of overlapping signals. DAPI (blue). Scale bars: 250 μm.
(TIF)

S11 Fig Tac2 overexpression in WT mice increases freezing responses to visual threat. (**A**) Schematic diagram of AAV-mCherry and AAV-Tac2 + AAV-mCherry delivery into the deep SC/PAG of WT mice. (**B**) Increased level of *Tac2* mRNA after overexpression (RT-qPCR; *n* = 2 mice/condition). (**C**) Schematic diagrams of the behavioral tests. (**D, E**) Representative images of mCherry expression (red, **D**) and Tac2 + mCherry co-expression (red, **E**). The sections were collected following the behavioral analysis. DAPI (blue). (**F, G**) Quantification of freezing responses (20.3 ± 4.9 s for mCherry, 44.1 ± 6.7 s for Tac2 + mCherry; *n* = 9 for mCherry and Tac2 + mCherry) and of the time spent in the brightly lit area (119.4 ± 15.8 s for mCherry, 132.3 ± 25.7 s for Tac2 + mCherry; *n* = 9 for mCherry and Tac2 + mCherry). Tac2 overexpression increased the total freezing time. No statistical difference was found during the light/dark exploration test. Unpaired two-tailed Student’s *t* test (mean ± SEM, *p* = 0.011 [*] for freezing time; *p* = 0.675 for time spent in the light). Scale bars: 250 μm. The data underlying this figure can be found in S1 Data.
(TIF)

S1 Table RNA-Seq alignment metrics. RNA seq was conducted with 3 controls and 4 *Brn3b* mutants. Table displays total reads, the percentage of unique reads, the percentage of unmapped reads, and the percentage of coding UTR.
(XLSX)

S2 Table The list of top 50 significantly down-regulated genes in mutants. (XLSX)

S3 Table The list of top 50 significantly up-regulated genes in mutants. (XLSX)

S1 Data Numerical values for all graphs in the main figures and supporting information. (XLSX)

## Abbreviations

AAV: adeno-associated virus
AP: alkaline phosphatase
CTB: cholera toxin B
dLGN: dorsal lateral geniculate nucleus
LP: lateral posterior nucleus
PAG: periaqueductal gray
RT-qPCR: reverse transcription-quantitative PCR
RGC: retinal ganglion cell
SC: superior colliculus
SGS: stratum griseum superficiale
SO: stratum opticum
TPM: transcripts per million
vLGN: ventral lateral geniculate nucleus
