# Supplementary figures and images for: Brn3b regulates the formation of fear-related midbrain circuits and defensive responses to visual threat
Source: PLoS Biol. 2023 Nov 20;21(11):e3002386. doi: 10.1371/journal.pbio.3002386 (PMC10695396; doi:10.1371/journal.pbio.3002386)

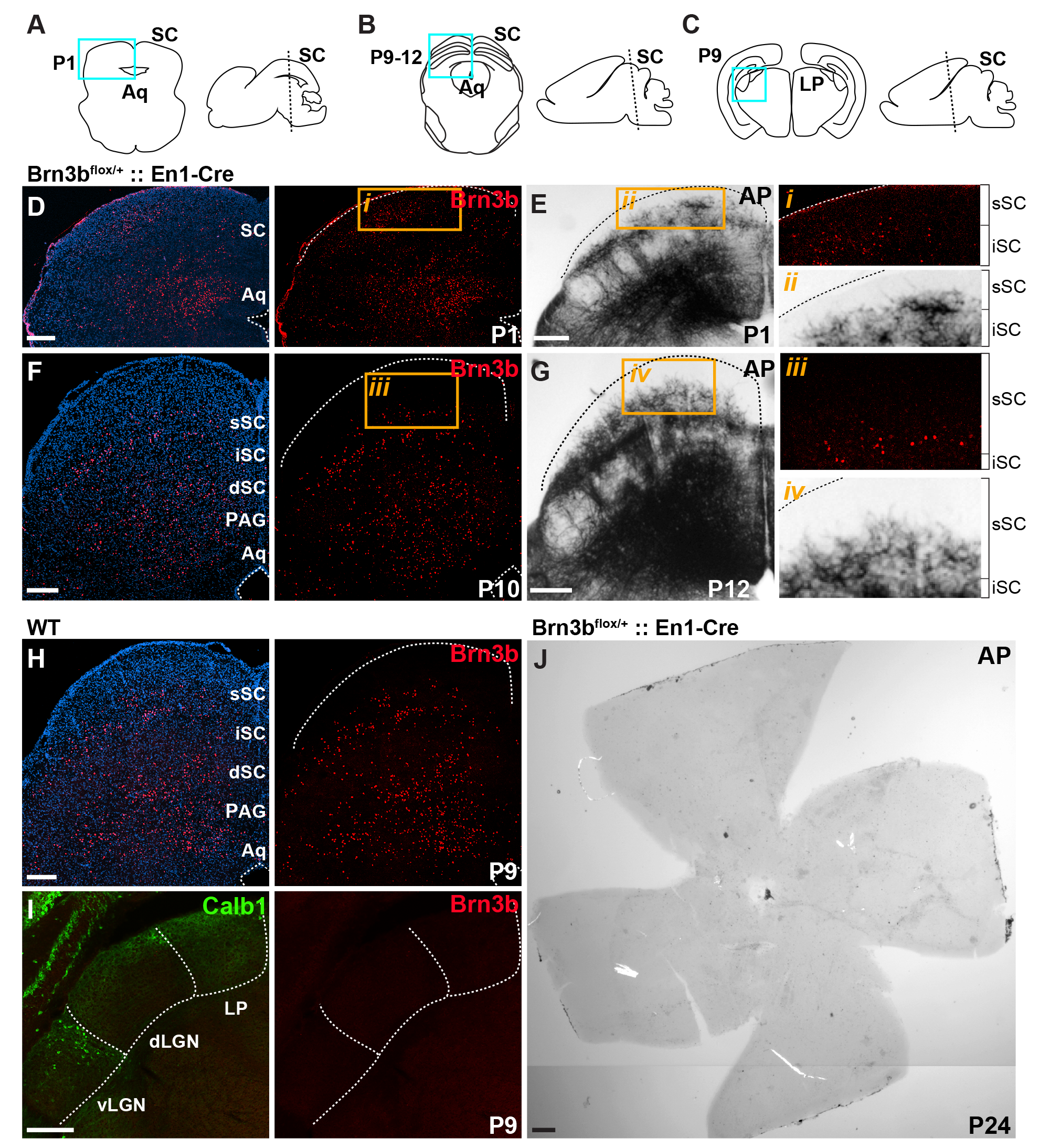

Supplement: S1 Fig — (A–C) Schematic diagrams of coronal images showing the brain area (boxed) used for analysis (left) and sagittal images depicting the level (dashed line) where the coronal section was obtained (right) at P1 (A) and P9-P12 (B and C). (D–G) Brn3b expression in Brn3bflox/+:: En1-Cre mouse, visualized by immunostaining at P1 (D) and P10 (F) and by AP labeling at P1 (E) and P12 (G). A magnified view of the boxed areas (i–iv) showed that Brn3b+ neurons are located close to the pia at P1 and become confined to the SO layer at P10/P12 (n = 3 mice/age/visualization method). Due to the poor quality of AP antibody staining and the required tissue clearing for AP staining, immunostaining and AP labeling were conducted using different sections/animals. (H, I) Brn3b immunostaining in WT mouse at P9. Brn3b expression is missing in the upper layer of superficial SC (SGS: H), consistent with observation in the Brn3bflox/+:: En1-Cre mice. No Brn3b signals were detected in LP (I). Immunostaining with anti-Brn3b was conducted at P9 because of very weak signals produced by Brn3b antibody beyond P12 (n = 3 mice). To demarcate dorsal lateral geniculate nucleus (dLGN) and LP in the thalamus, calbindin antibody (green) was used (Grubb and colleagues [57]). (J) No AP signals were detected in the retina of Brn3bflox/+:: En1-Cre mouse (n = 3 animals). Dashed lines indicate either the pia surface or boundary of dLGN and LP (D–I). Blue (DAPI). Scale bar: 200 μm. (TIF) [file pbio.3002386.s001.tif]

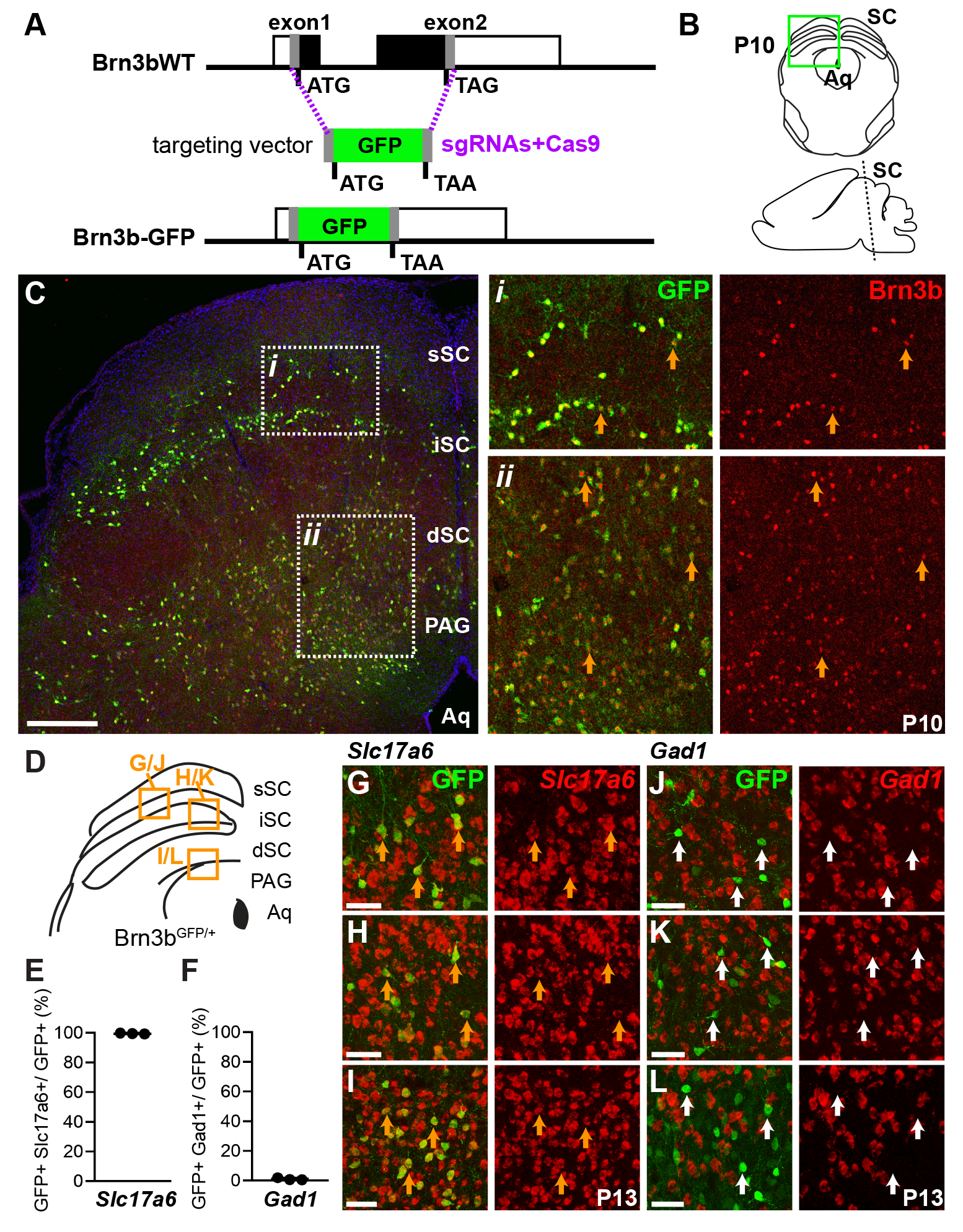

Supplement: S2 Fig — (A) CRISPR/Cas9-mediated genome-editing strategy to generate Brn3bGFP/+ (Brn3b-GFP) knock-in mouse line. Two sgRNAs (for 5′ and 3′ ends) were utilized. The targeting vector contains the GFP with a small t intron flanked by homology arms (indicated by gray boxes) to 162 bp upstream and 100 bp downstream of Brn3b open reading frame. This strategy produces a mouse in which the GFP sequence replaces an open reading frame of Brn3b. (B) Schematic diagrams of a coronal image showing the brain area (boxed) used for analysis (top) and a sagittal image depicting the level (dashed line) where the coronal section was obtained (bottom). (C) (Left) A section of Brn3bGFP/+ mouse brain labeled with antibodies to GFP (green) and Brn3b (red) at P10. (Right) Magnified view of the boxed areas (i, ii). Quantification revealed that approximately 95% of Brn3b+ cells express GFP (1,178.8 ± 41.6 cells/animal, n = 4 animals) and approximately 93% of GFP+ cells express Brn3b (1,189.0 ± 43.1 cells/animal, n = 4 animals), confirming that GFP+ cells faithfully represent Brn3b+ neurons. Orange arrows indicate examples of overlapping signals. DAPI (blue). (D) Schematic diagram showing the brain areas of the Brn3bGFP/+ mouse analyzed using double labeling by in situ hybridization with probes to Slc17a6 and Gad1 and immunostaining with anti-GFP antibody. (E–L) Brn3b+ neurons (green, visualized by GFP antibody) express Slc17a6 (red, G–I) but not Gad1 (red, J–L). Quantification of overlap between Slc17a6 and GFP (E: 834.7 ± 47.7 cells/animal, n = 3 animals) or between Gad1 and GFP (F: 861.7 ± 17.9 cells/animal, n = 3 animals). Orange arrows indicate examples of overlapping signals; white arrows indicate examples of non-overlapping signals. Scale bars: 250 μm (C), 50 μm (G–L). The data underlying this figure can be found in S1 Data. (TIF) [file pbio.3002386.s002.tif]

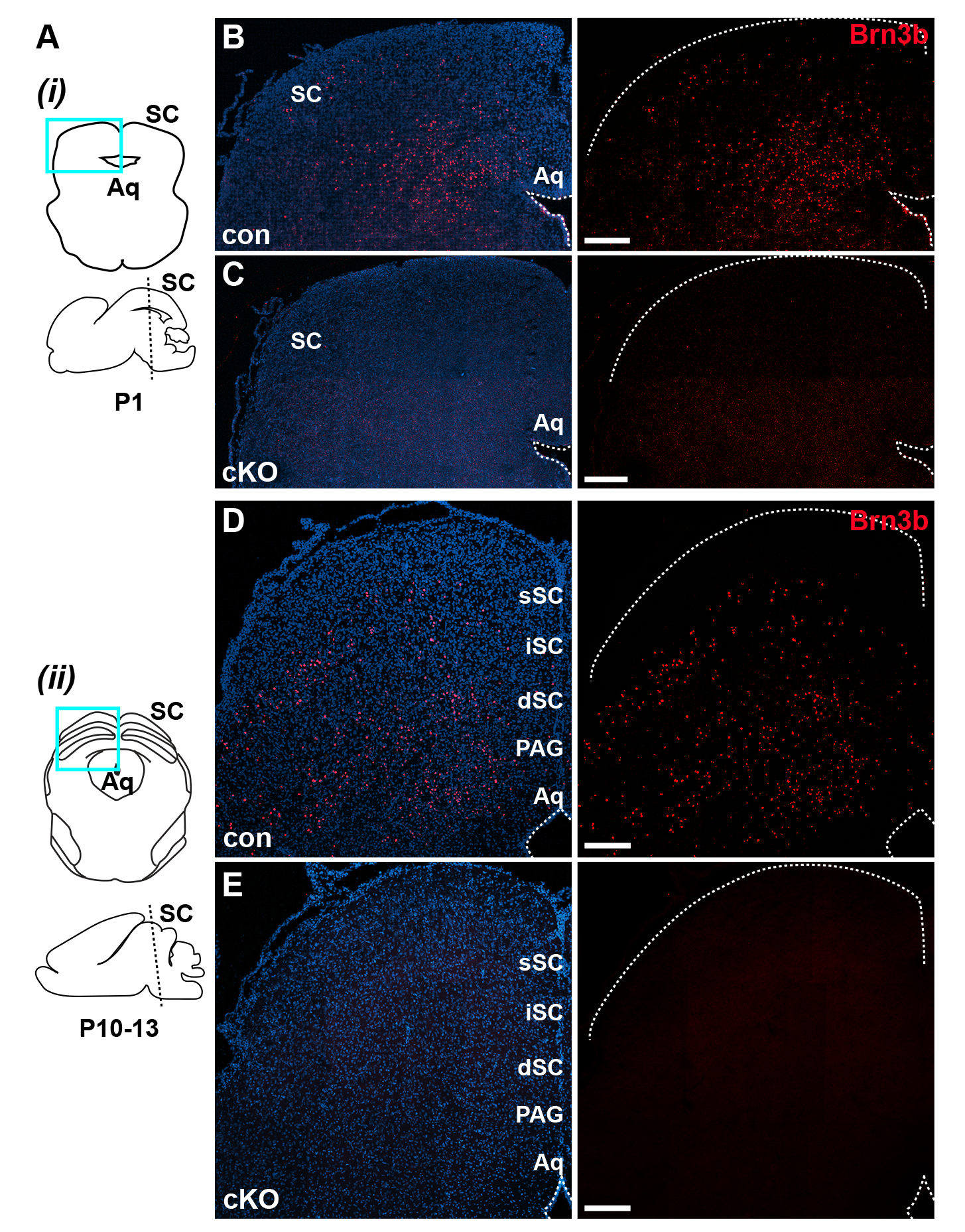

Supplement: S3 Fig — (A) Schematic diagrams showing the brain area (boxed) of a coronal section used for analysis (top) and a sagittal diagram depicting the level (dashed line) where such coronal section was obtained (bottom) at P1 (i) and at P10-P13 (ii). Brn3b expression (red) in control (B and D) and cKO (C and E) brains, visualized by immunostaining (n = 3 mice/group/development stage). DAPI (blue). Scale bars: 200 μm. (TIF) [file pbio.3002386.s003.tif]

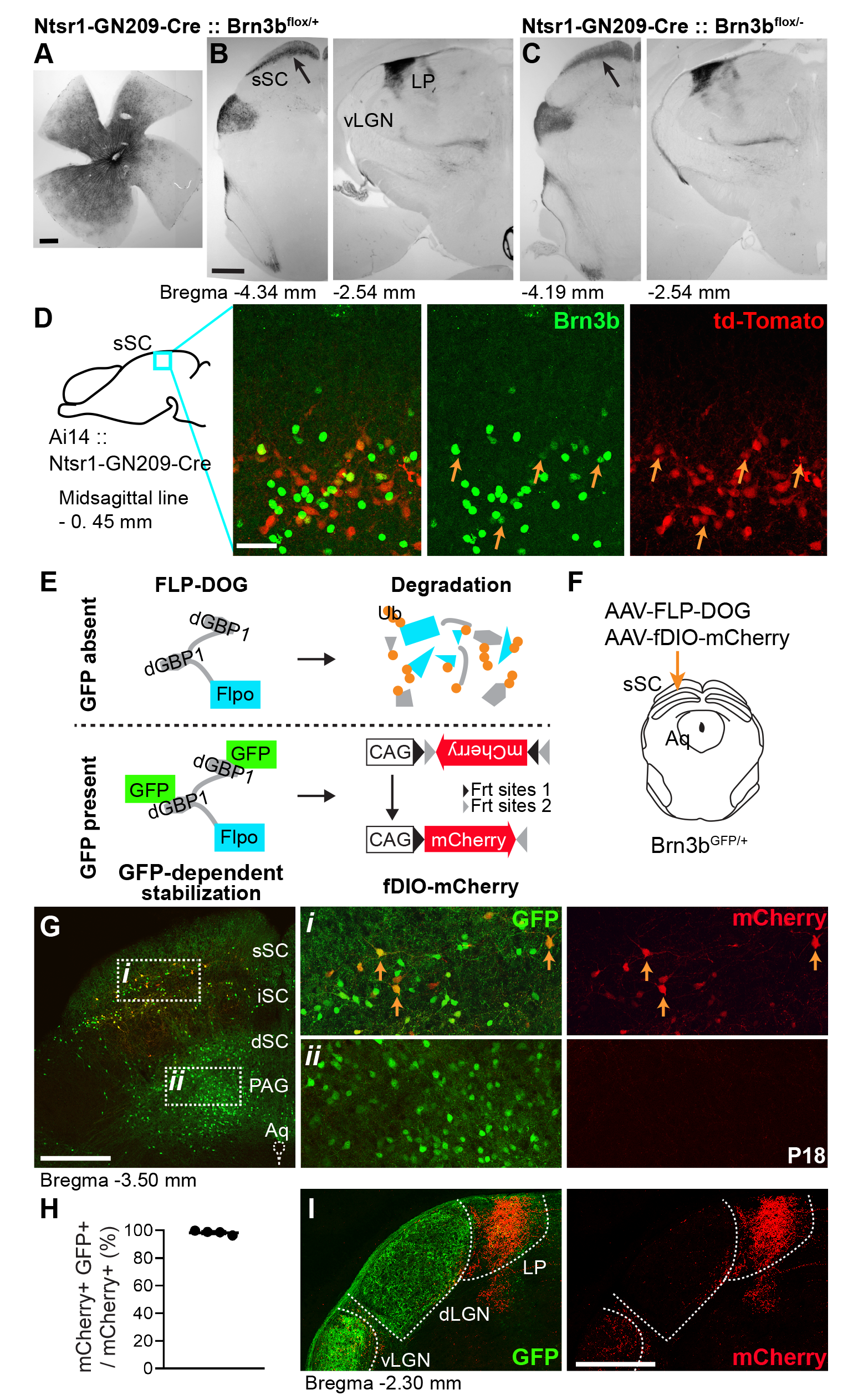

Supplement: S4 Fig — (A–D) Ntsr1-GN209-Cre line expresses Cre in the superficial SC neurons projecting to LP (Gale and Murphy [27]). (A) The retina of Ntsr1-GN209-Cre:: Brn3bflox/+ mouse showing AP signals. (B) Brn3b+ neurons in superficial SC (left, arrow) and projections to LP (right), visualized by AP signals in control brain (Ntsr1-GN209-Cre:: Brn3bflox/+). (C) No structural changes were detected in the mutants (Ntsr1-GN209-Cre:: Brn3bflox/-). To ensure that the labeled axons in the LP originated from the SC, and not the retina, both eyes were enucleated. Additional AP covered areas were detected in the midbrain, suggesting that Cre expression pattern in the Ntsr1-GN209-Cre line differs from the pattern in En1-Cre (n = 3 mice). (D) (Left) Schematic diagram showing the brain area analyzed to examine Brn3b expression in Ntsr1-GN209-Cre:: Ai14 mice expressing Cre-dependent td-Tomato. (Right) Overlap between Brn3b (green) and td-Tomato (red). Quantification revealed that approximately 23% of td-Tomato+ cells were Brn3b+ (604.3 ± 41.3 cells/animal, n = 3 animals). Arrows indicate examples of overlapping signals. (E) Schematic diagram depicting the FLP-DOG/ fDIO-mCherry strategy. FLP-DOG is unstable and degrades in the absence of GFP. Binding of GFP stabilizes FLP-DOG, which coverts fDIO-mCherry to an active form resulting in mCherry expression (Flpo, codon-optimized FLP; dGBP1, destabilized GFP-binding protein; Ub, ubiquitin molecules; CAG, promoter). (F) Schematic diagram of AAV-FLP-DOG and AAV-fDIO-mCherry delivery into the SC of Brn3bGFP/+ mouse. (G) Representative image of mCherry expression in the SC. Magnified view of the boxed areas showing overlap between mCherry+ and GFP+ (i.e., Brn3b+ cells) in superficial SC (i) but no mCherry signals in deep SC/PAG (ii). Arrows indicate examples of overlapping signals. (H) Quantification revealed that approximately 98% of mCherry+ cells express GFP (947 cells, n = 4 animals). (I) Projections of mCherry+ axons to LP. Dense GFP labeling in dLGN [file pbio.3002386.s004.tif]

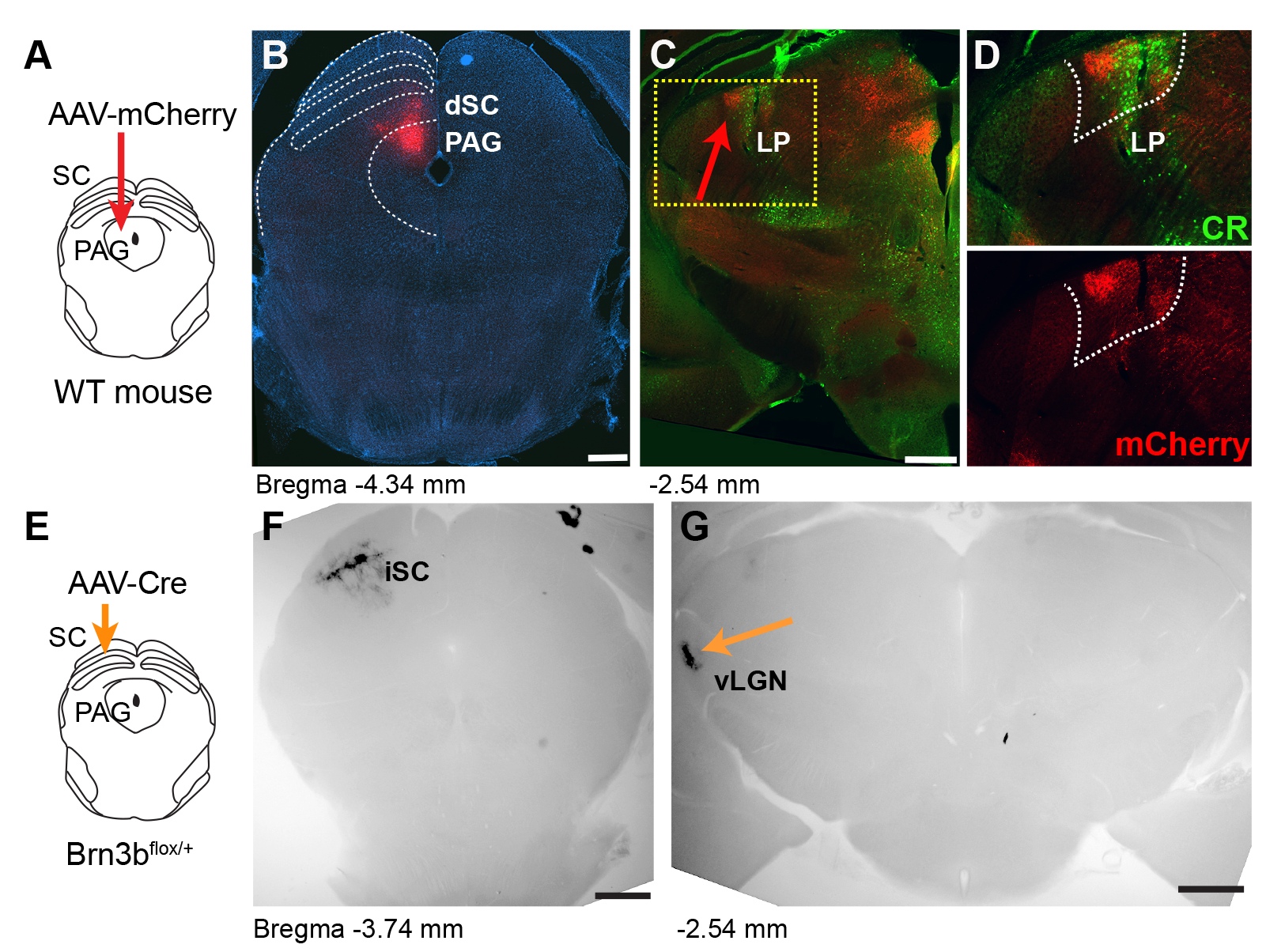

Supplement: S5 Fig — (A) Schematic diagram of AAV-mCherry injection into deep SC/PAG. (B–D) Representative images showing mCherry expression in deep SC/PAG (B) and mCherry+ neuronal projections to LP (arrow in C). Magnified view of the boxed area (D). LP was identified by immunostaining with calretinin (CR) antibody (Byun and colleagues [22]) (n = 3 mice; DAPI (blue)). (E) Schematic diagram of AAV-Cre injection into intermediate SC of Brn3bflox/+ mouse. (F, G) Representative images of AP signals in intermediate SC (E) and projections to ventral LGN (arrow in G), indicating that Brn3b+ neurons in the intermediate SC project to ventral LGN (n = 2 mice). Very faint signals in LP (G) might originate from neurons in other layers of the dorsal midbrain. However, if other layers beyond the targeted areas were obviously labeled by the AAV, such animals were excluded from analysis (A–G). Scale bars: 500 μm. (TIF) [file pbio.3002386.s005.tif]

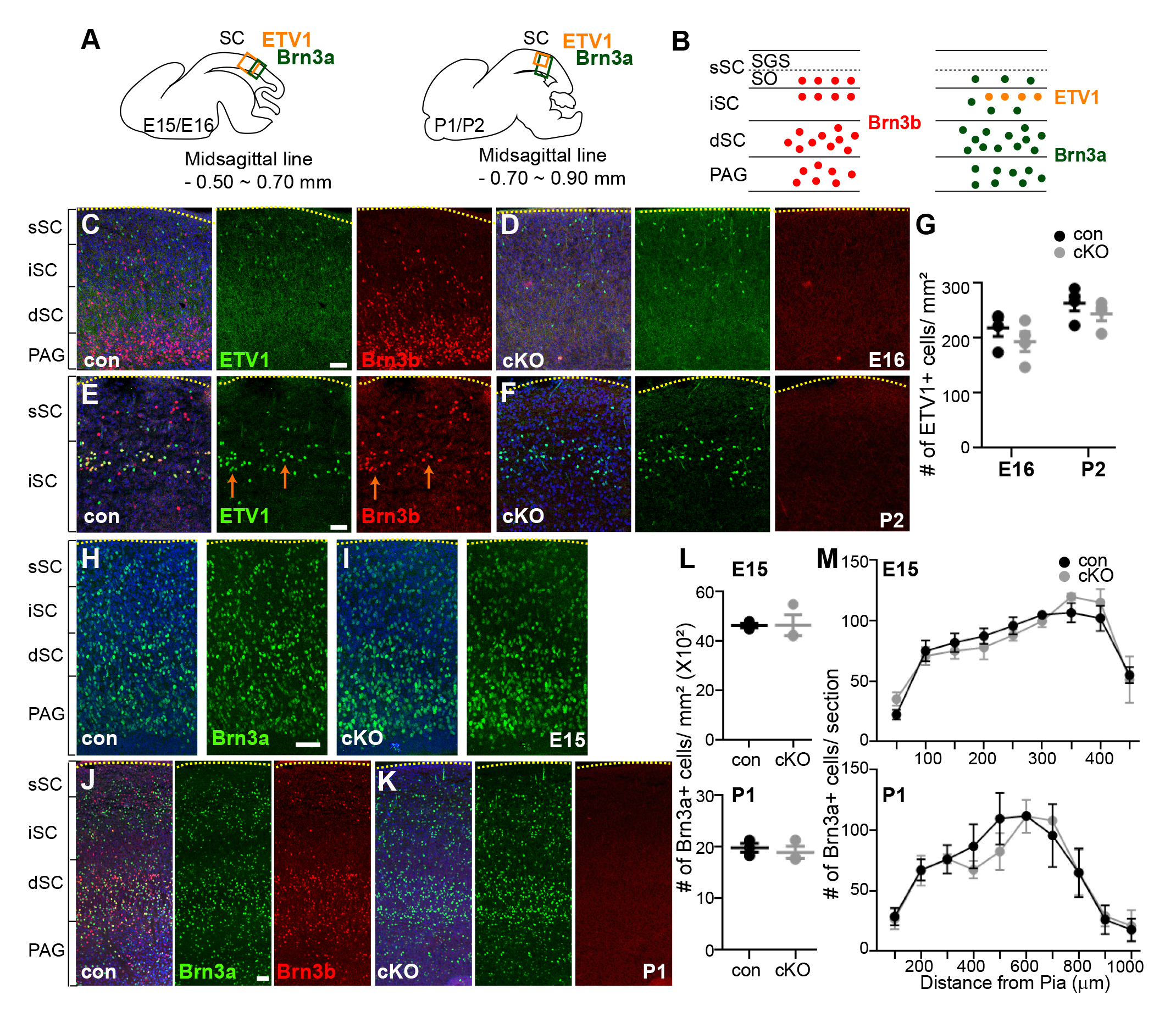

Supplement: S6 Fig — (A, B) Schematic diagram illustrating the level of the sagittal sections as a lateral distance from the midsagittal line at E15, E16, P1, and P2 (A) and the layer distribution of Brn3b+ versus ETV1+ neurons and Brn3b+ versus Brn3a+ neurons in the dorsal midbrain (B). (C–F) Sections stained for Brn3b (red) and ETV1 (green) at E16 (C, D) and P2 (E, F). Neither Brn3b expression nor ETV1+/ Brn3b+ neurons in superficial SC were detected at E16 in control (C). Segregation of Brn3b+ neurons into superficial SC and intermediate SC was detectable at P2 in control (E). Some Brn3b+ neurons in the intermediate SC but not in superficial SC express ETV1 (arrows). (G) Quantification of ETV1+ neurons shows no clear difference between control and cKO (218.5 ± 15.4/ mm2 for control, 193.8 ± 17.8/ mm2 for cKO at E16, 263.8 ± 14.4/mm2 for control, 244.3 ± 12.4/ mm2 for cKO at P2; n = 4 mice/group/developmental stage). Unpaired two-tailed Student’s t test (mean ± SEM; p = 0.333 at E16; p = 0.345 at P2). (H–K) Sections stained for Brn3b (red) and Brn3a (green) at E15 (H, I) and P1 (J, K). No double Brn3b/Brn3a staining was conducted at E15 due to incompatibility of Brn3b and Brn3a antibodies (both raised in goat). Mouse antibody to Brn3a, used at P1, did not produce any signals at E15, likely due to a low level of Brn3a at this stage. (L, M) Quantification of Brn3a+ neuronal number and position at E15 and P1 showed no obvious difference between control and cKO (4,641.9 ± 96.2/mm2 for control, 4,656.7 ± 421.4/mm2 for cKO at E15, 1,985.5 ± 85.4/mm2 for control, 1,898.5 ± 118.2/mm2 for cKO at P1; n = 3 mice/group/developmental stage). Unpaired two-tailed Student’s t test (mean ± SEM; p = 0.974 at E15; p = 0.583 at P1). The dashed lines delineate the pial surface (C–F and H–K). DAPI (blue). Scale bars: 50 μm. The data underlying this figure can be found in S1 Data. (TIF) [file pbio.3002386.s006.tif]

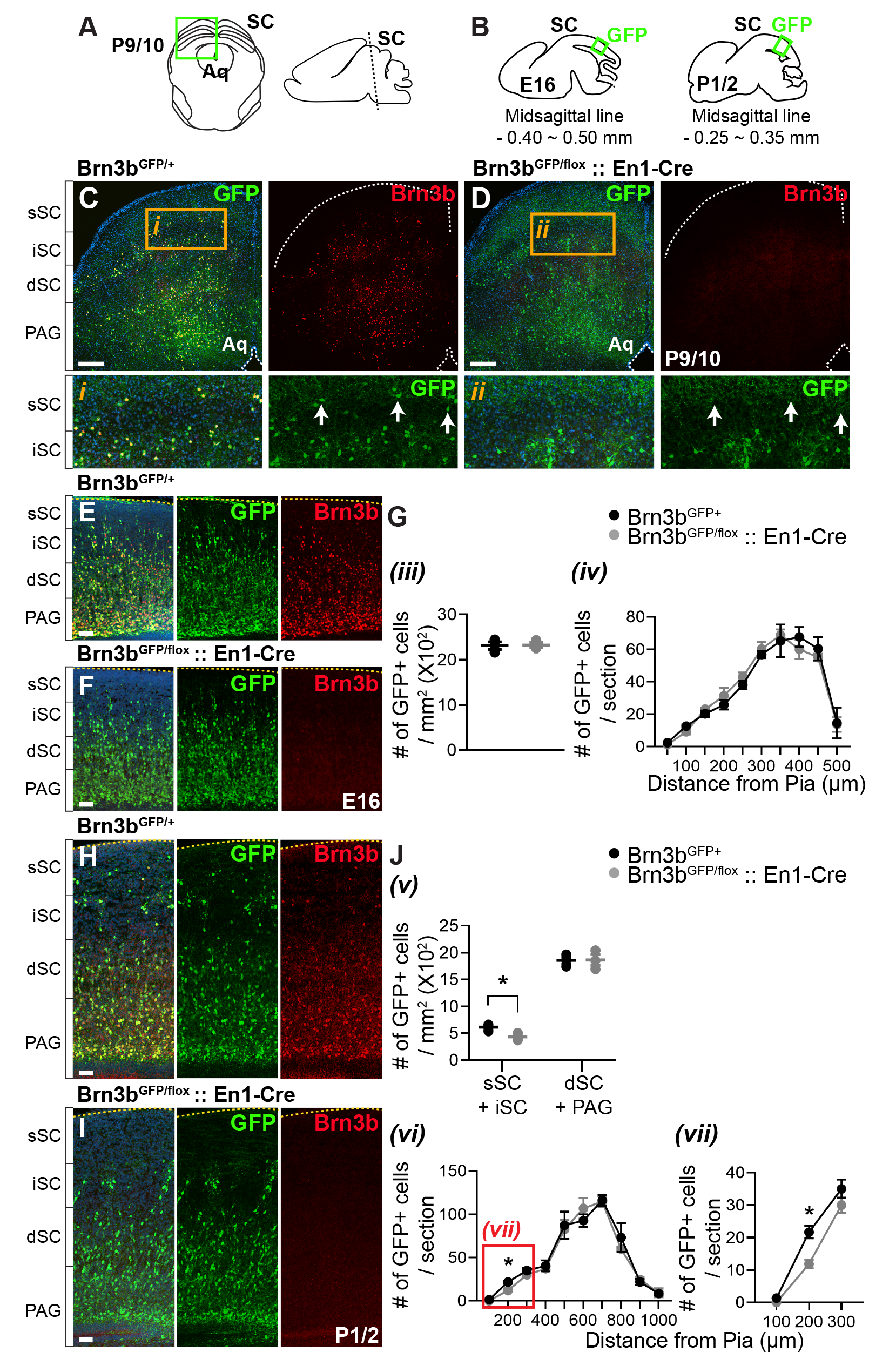

Supplement: S7 Fig — (A) Schematic diagram of a coronal image showing the brain areas (boxed) used for analysis (left) and a sagittal image depicting the level (dashed line) where a coronal section was obtained (right) at P9/P10. (B) Schematic diagram illustrating the level of the sagittal sections as lateral distances from the midsagittal line at E16 and P1/2. (C, D) Double immunostaining with anti-Brn3b (red) and anti-GFP (green) shows no Brn3b expression in the Brn3bGFP/flox:: En1-Cre at P9/P10. Magnified view of the boxed areas showing the loss of GFP+ (i.e., Brn3b+) neurons in the superficial SC (i, ii), indicated by arrows (n = 3 animals/genotype). DAPI (blue). (E, F) Representative images of sections stained for Brn3b (red) and GFP (green) at E16. DAPI (blue). (G) Quantification of GFP+ neurons (iii) and their distribution (iv) showed no obvious differences at E16 (2,306.9 ± 83.7/mm2 for Brn3b GFP/+, 2,319.6 ± 56.6/mm2 for Brn3b GFP/flox:: En1-Cre, n = 3 animals/genotype). Unpaired two-tailed Student’s t test (mean ± SEM; p = 0. 906). (H, I) Representative images of sections stained for Brn3b (red) and GFP (green) at P1/2. DAPI (blue). (J) Quantification of GFP+ neurons (v) and their distribution (vi) revealed that Brn3b loss decreases the number of neurons in the superficial and intermediate layers (613.6 ± 37.8/mm2 for Brn3b GFP/+, 431.3 ± 39.6/mm2 for Brn3b GFP/flox:: En1-Cre, n = 3 animals/genotype). Superficial and intermediate layers were defined as located within 350 μm from the pia. Unpaired two-tailed Student’s t test (mean ± SEM; p = 0.029 [*] for the cell number and p = 0.013 [*] for the distribution). Magnified view of the boxed area (vii). No clear difference was detected in the deep SC/PAG (1,857.1 ± 65.4/mm2 for Brn3b GFP/+, 1,863.0 ± 101.0/mm2 for Brn3b GFP/flox:: En1-Cre, n = 3 animals/genotype). Unpaired two-tailed Student’s t test (mean ± SEM; p = 0. 964). The dashed lines delineate the pial surface (C–F and H–I). DAPI (blue). Scale bars: 200 μm (C–D) and 50 μm [file pbio.3002386.s007.tif]

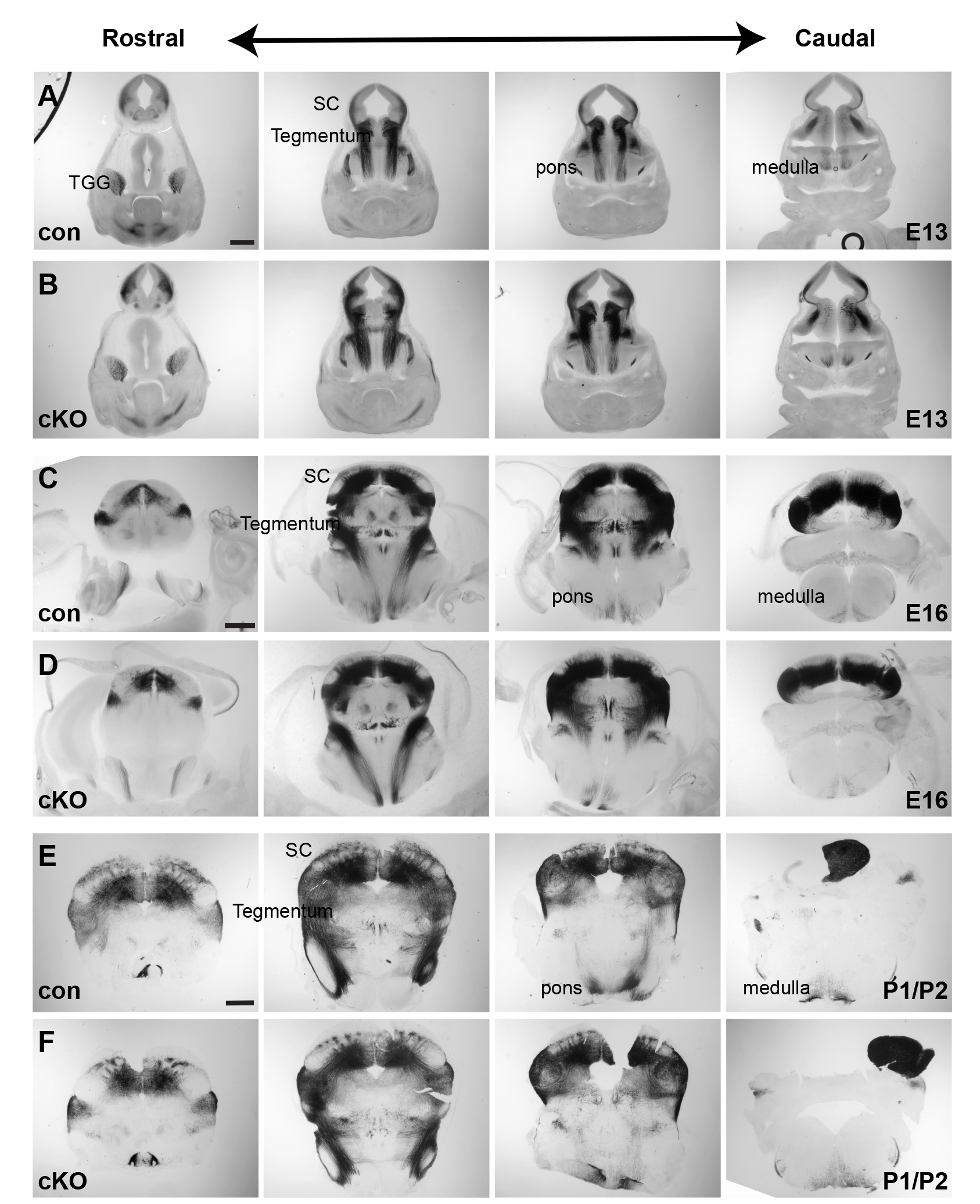

Supplement: S8 Fig — Neuronal projections were visualized by AP signals at E13 (A, B), E16 (C, D), and P1/P2 (E, F). Projections to the ventral midbrain, including tegmentum, pons, and medulla, were detected during embryonic development and disappeared after birth. Overall, no differences were found between control and cKO (n = 3 mice/group/developmental stage). The images were taken using consecutive sections covering the entire superior colliculus, 200 μm apart at E13, 200 μm apart at E16, and 600 μm apart at P1/P2. Scale bars: 500 μm. (TIF) [file pbio.3002386.s008.tif]

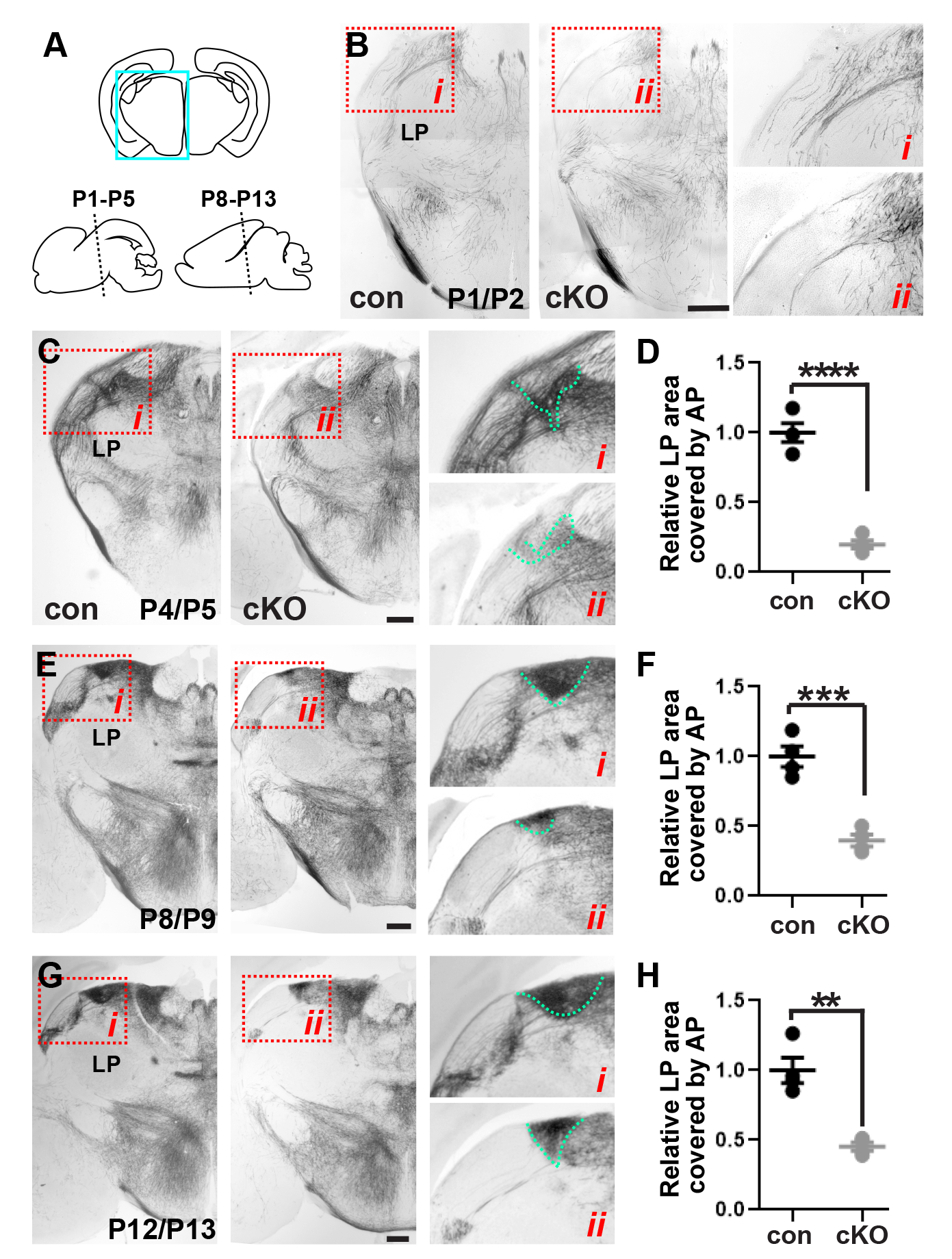

Supplement: S9 Fig — Axonal projections were visualized by AP signals. (A) Schematic diagram of a coronal image showing the brain area (boxed) used for analysis (top) and sagittal images depicting the level (dashed line) where each coronal section was obtained (bottom) at P1-P5 and P8-P13. (B) (Left) Axonal branches in LP were barely detectable at P1/P2 in control and cKO mice (n = 4 mice/group). (Right) Magnified view of the boxed areas. (C) (Left) Axonal branches in LP were clearly noticeable at P4/P5 in control but barely visible in cKO mice (n = 4 mice/group). (Right) Magnified view of the boxed areas showing the LP regions used for analysis. (D) Quantification was presented as a relative difference between the AP-covered LP area in controls and mutants (1.00 ± 0.07 for control, 0.20 ± 0.03 for cKO; n = 4 mice/group). (E) (Left) Axonal branches in LP became abundant at P8/P9 in control and cKO mice, but the LP area covered by AP signals was smaller in cKO (n = 4 mice/group). (Right) Magnified view of the boxed areas. (F) Quantification (1.00 ± 0.07 for control, 0.40 ± 0.04 for cKO). (G) (Left) Axonal innervation in the LP is complete at P12/P13 in control mice, however, clearly decreased in cKO animals (n = 4 mice/group). (Right) Magnified view of the boxed areas. (H) Quantification (1.00 ± 0.09 for control, 0.45 ± 0.03 for cKO); unpaired two-tailed Student’s t test (mean ± SEM; p < 0.0001 [****] at P4/P5; p = 0.0004 [***] at P8/P9; p = 0.0012 [**] at P12/P13). Scale bars: 250 μm. The data underlying this figure can be found in S1 Data. (TIF) [file pbio.3002386.s009.tif]

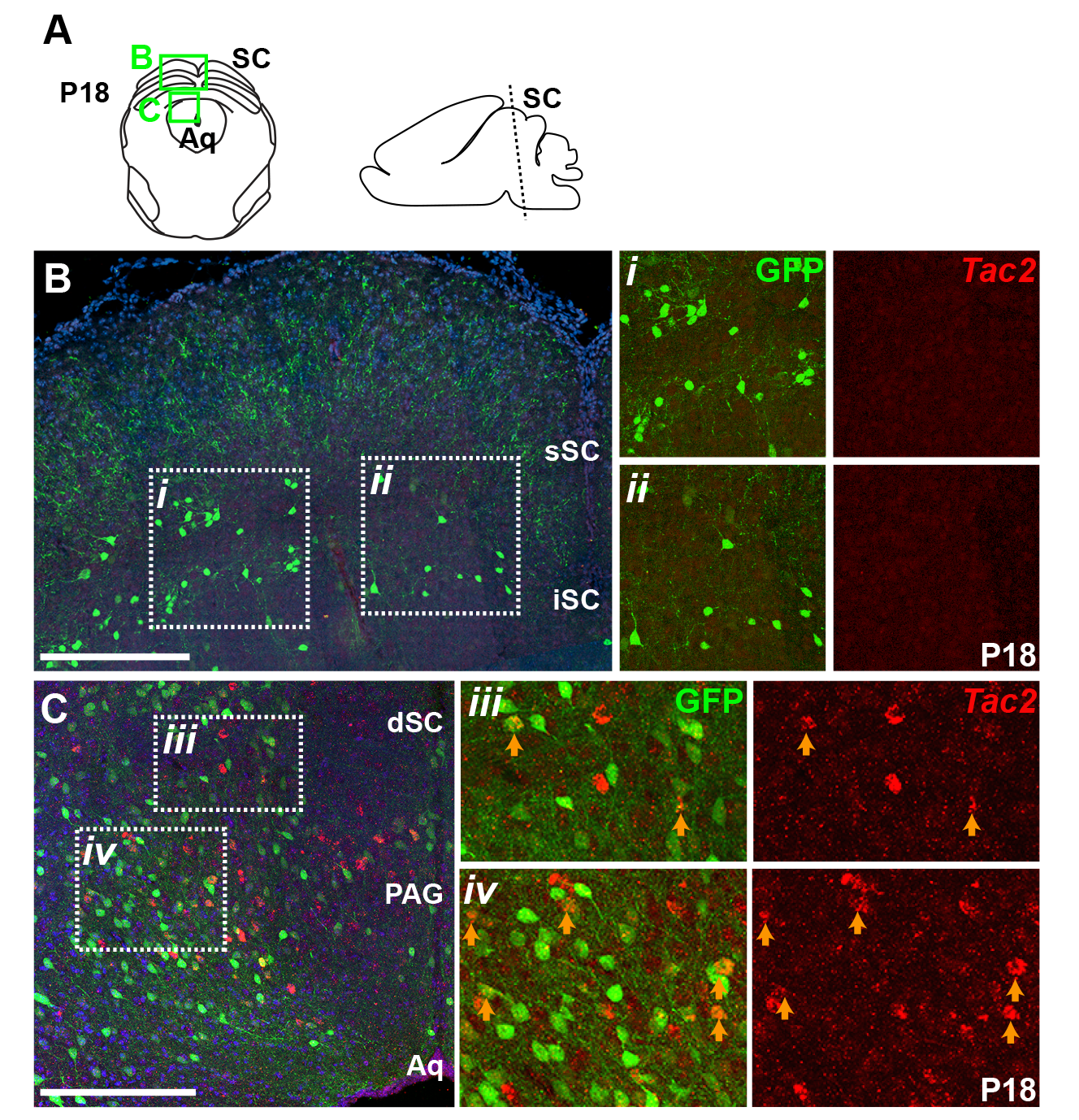

Supplement: S10 Fig — (A) Schematic diagrams of a coronal image showing the brain areas (boxed) used for analysis (left) and a sagittal image depicting the level (dashed line) where each coronal section was obtained (right). (B, C) Brain sections of Brn3bGFP/+ mouse labeled with anti-GFP (i.e., Brn3b; green) and in situ probe to Tac2 (red) at P18. (B) (Left) No Tac2+ signals were detected in the superficial and intermediate SC layers. (Right) Magnified view of the boxed areas (i, ii). (C) (Left) Tac2+ cells are detectable in deep SC/PAG. (Right) Magnified view of the boxed areas (iii, iv). Quantification revealed that approximately 57% of Tac2+ cells are Brn3b+ (70.7 ± 7.2 cells/animal, n = 3 animals). Arrows indicate examples of overlapping signals. DAPI (blue). Scale bars: 250 μm. (TIF) [file pbio.3002386.s010.tif]

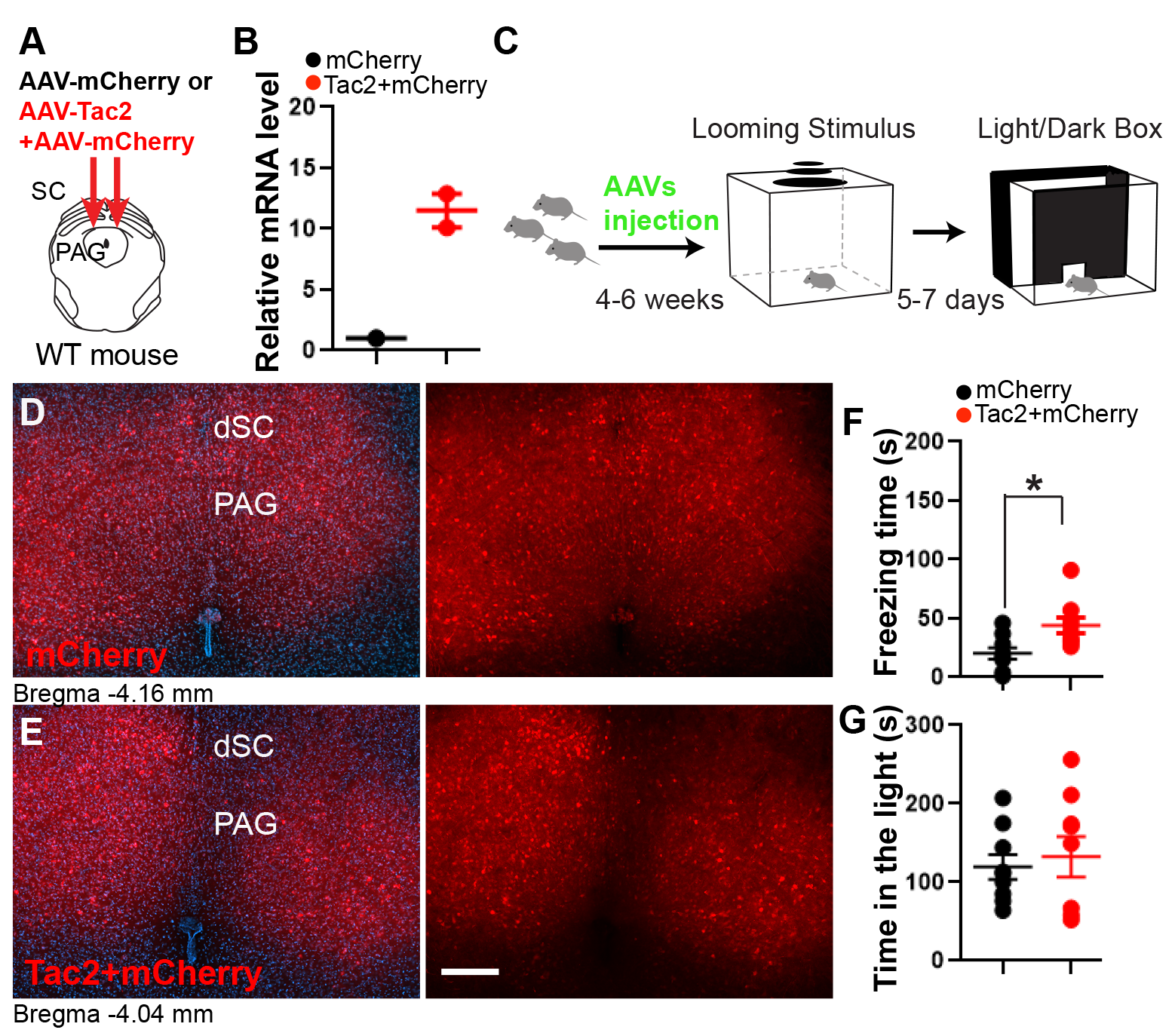

Supplement: S11 Fig — (A) Schematic diagram of AAV-mCherry and AAV-Tac2 + AAV-mCherry delivery into the deep SC/PAG of WT mice. (B) Increased level of Tac2 mRNA after overexpression (RT-qPCR; n = 2 mice/condition). (C) Schematic diagrams of the behavioral tests. (D, E) Representative images of mCherry expression (red, D) and Tac2 + mCherry co-expression (red, E). The sections were collected following the behavioral analysis. DAPI (blue). (F, G) Quantification of freezing responses (20.3 ± 4.9 s for mCherry, 44.1 ± 6.7 s for Tac2 + mCherry; n = 9 for mCherry and Tac2 + mCherry) and of the time spent in the brightly lit area (119.4 ± 15.8 s for mCherry, 132.3 ± 25.7 s for Tac2 + mCherry; n = 9 for mCherry and Tac2 + mCherry). Tac2 overexpression increased the total freezing time. No statistical difference was found during the light/dark exploration test. Unpaired two-tailed Student’s t test (mean ± SEM, p = 0.011 [*] for freezing time; p = 0.675 for time spent in the light). Scale bars: 250 μm. The data underlying this figure can be found in S1 Data. (TIF) [file pbio.3002386.s011.tif]
